# Backbone Torsion Angle Determination Using Proton Detected Magic-Angle Spinning Nuclear Magnetic Resonance

**DOI:** 10.1021/acs.jpclett.1c03267

**Published:** 2021-12-27

**Authors:** Kai Xue, Evgeny Nimerovsky, Kumar A. Tekwani Movellan, Stefan Becker, Loren B. Andreas

**Affiliations:** Max Planck Institute for Biophysical Chemistry, Department of NMR Based Structural Biology, Am Fassberg. 11, 37077 Goettingen, Germany

## Abstract

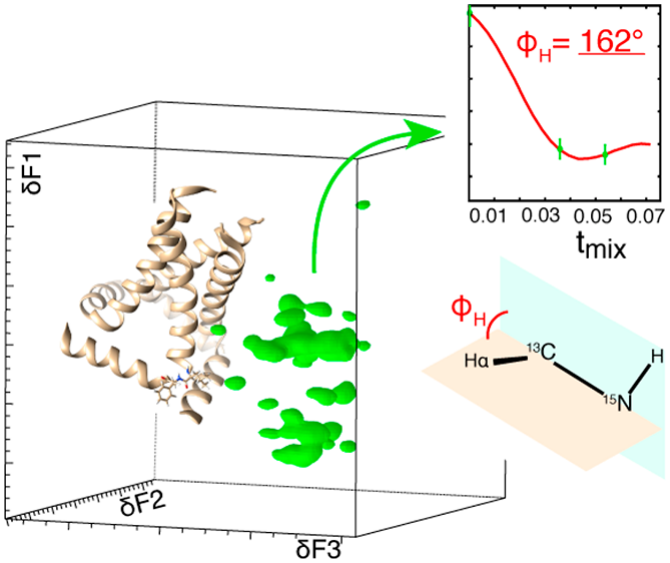

Protein torsion angles define the backbone secondary structure of proteins. Magic-angle spinning (MAS) NMR methods using carbon detection have been developed to measure torsion angles by determining the relative orientation between two anisotropic interactions—dipolar coupling or chemical shift anisotropy. Here we report a new proton-detection based method to determine the backbone torsion angle by recoupling NH and CH dipolar couplings within the HCANH pulse sequence, for protonated or partly deuterated samples. We demonstrate the efficiency and precision of the method with microcrystalline chicken α spectrin SH3 protein and the influenza A matrix 2 (M2) membrane protein, using 55 or 90 kHz MAS. For M2, pseudo-4D data detect a turn between transmembrane and amphipathic helices.

Protein function is closely related to secondary, tertiary, and quaternary structure. The two peptide backbone torsion angles φ and ψ represent one way of defining the protein fold.^1,2^ In the solid state, the selective recoupling of two orientation-dependent tensors such as CSA and dipolar interactions leads to, when suitably selected, measurement of φ, ψ, or side-chain angles.^3,4^ In solution, torsion angle restraints are usually inferred from J-couplings or measured with cross-correlated relaxation.^5−10^ Previous methods to determine torsion angles in solids have been developed based on detection of carbon or nitrogen at moderate magic-angle spinning (MAS) frequencies of ∼20 kHz and below.^11−15^ They are effective for exceptionally well-resolved spectra typical of protein microcrystals but are difficult to implement for large proteins or membrane proteins due to a reduction in sensitivity and resolution for such systems. The result is that most contemporary protein structure determination studies employ empirical NMR methods such as TALOS,^16−20^ which is based on a large repository of experimental chemical shifts. TALOS is widely used to predict torsion angles, and the result can be relied upon to identify regular secondary structure, because these local structures are typically well sampled in previously determined structures. Despite the overwhelming success of TALOS in structure determinations, discrepancies do occasionally occur between X-ray crystal structures and TALOS predictions, mostly in loops. This motivates direct experimental methods for measurement of backbone torsion angles, particularly for glycine residues that lack a β carbon, which is strongly considered in TALOS.

Thanks to the development of solid-state NMR hardware at high field and faster MAS, as well as tailored deuterium labeling, the inherent sensitivity of proton detection is more and more routinely exploited for biomolecular structures.^21−26^ New assignment pulse sequences were proposed based on proton detection,^24,27,28^ and several methods have also been proposed to determine H–X dipolar couplings^29−33^ as well as long-range H–X^34,35^ and H–H distance restraints.^36−38^ However, solid-state NMR methods for backbone torsion angle determination in the current ultrafast MAS regime (MAS frequency of about 60 to 120 kHz) with proton detection are, to our knowledge, as yet undeveloped. The extension to the ultrafast MAS regime promises improved performance via proton detection, if suitable pulse sequences can be developed. The previously reported rotor synchronized pulse sequences demand high power pulses, which are multiples of the spinning frequency and cannot be readily implemented in contemporary ultrafast MAS probes.

We solve this problem using pseudo-3D and pseudo-4D spectra based on the HCANH signal transfer pathway to determine dipolar coupling and torsion angles in microcrystalline SH3 and the membrane protein influenza A M2. In combination with a recently developed deuterium labeling scheme,^39^ we show that the torsion angle can also be reliably determined for glycine, which lacks a side chain.

The dipolar recoupling element we use here is a recently reported MODifiEd RN (MODERN) scheme.^40^ The MODERN scheme has a reasonable power level requirement and good scaling factors for dipolar couplings and can be applied in ultrafast MAS.^40^ Another important feature of this sequence is that radio frequency (RF) power missetting introduces intensity variations rather than a change in the dipolar scaling factor. It makes extraction of the dipolar coupling more straightforward than is the case for most R-symmetry based sequences.^40,41^ This feature enables accurate calibration of proton power levels during dipolar recoupling.

We recorded ^1^H-detected (H)(CA)NH-MODERN spectra at a MAS frequency of 55.555 kHz (1.3 mm Bruker rotor), employing cross-polarization (CP)^42^ for polarization transfer. We used a partly deuterated “alpha proton exchange by transamination” (α-PET) labeled sample^39^ of the α-spectrin SH3 domain and a uniformly labeled [^13^C, ^15^N]-SH3 sample. In the α-PET labeling scheme, Hα is introduced for 13 amino acids in a highly deuterated background. Experimental results were fitted with numerical simulations using in-house MATLAB scripts (details in the Supporting Information).

Figure 1 shows the pulse sequence used to correlate two dipolar coupling vectors, H–N and H–Cα, via simultaneous incrementation of the recoupling time in both periods in dashed rectangles. These vectors define the torsion angle φ_H_, which is shifted by −60° from the torsion angle φ.^4^ Dipolar coupling is reintroduced using a modified R-symmetry (MODERN) sequence. MODERN was previously reported to minimize H–H homogeneous interactions and maximize the recoupled heteronuclear dipolar coupling strength. Considering the power limitation of the probe, we chose MODER5.^40^ Approximately 137 kHz proton power was applied for 55.555 kHz spinning. Benefiting from the favorable scaling factor (*K*_sc_) from this sequence (∼0.5), only several hundred microseconds of recoupling are needed to observe HN and HC dipolar oscillations and encode HN–HC torsion angles. As described previously and shown with the simulations in Figure S1, the MODER5 dipolar oscillation curve is sensitive to RF inhomogeneity. The impact of an RF misset is to attenuate the amplitudes in dipolar oscillations. This feature can be used to calibrate the optimal power level setting by observing minima in signal intensities after a certain recoupling period (Figure S2). To account for the influence of RF-field inhomogeneity in simulation, we assumed that the B1 field has a Gaussian distribution.^54−56^ The detailed fitting procedure considering RF inhomogeneity and ^13^C/^15^N T_2,eff_ decay is described in Figure S3. Since the angle determined from dipolar recoupling signals is a projection angle, a transformation is needed to convert the projection angle to the torsion angle (eq S5 in the SI). For transformation, two input bond angles are used—θ_NCαHα_ = 71° and θ_HNCα_ = 120°. Figure 1C shows the schematic representation of the torsion angle, determined by two planes, and the projection angle, determined by two vectors.

**Figure 1:**
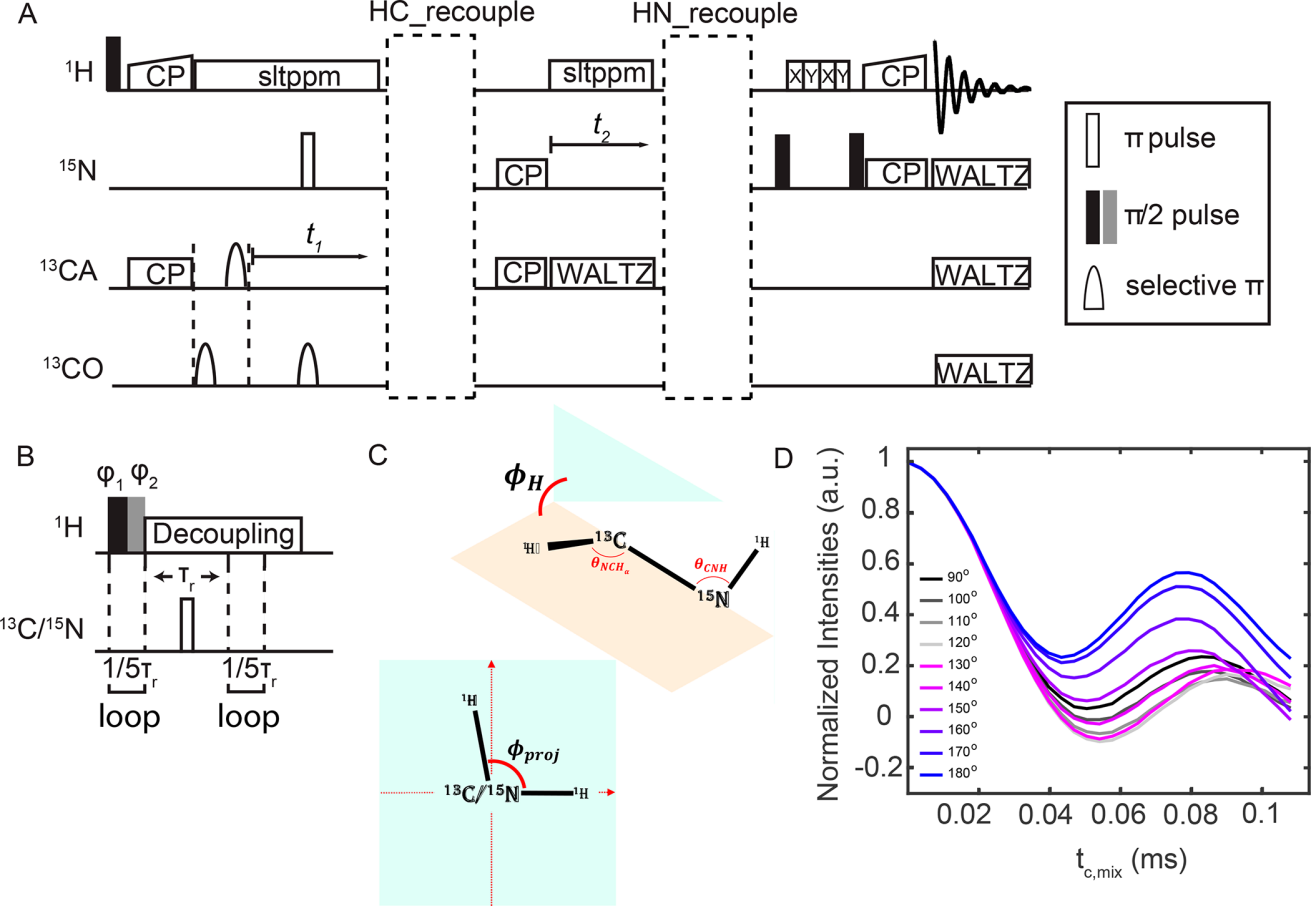
Torsion angle determination using MODER5. (A, B) Pulse sequence diagram for the torsion angle measurement. The MODER5 recoupling element shown in (B) occurs in each of the dashed rectangular regions in (A). MODER5 consists of two π/2 pulses as the basic element. The length of each π/2 pulse equals 1/10 of the rotor period and pulse phase φ_1_= 36°, φ_2_ = 164.2°. Following recoupling, a Hahn echo period is used to refocus the nitrogen or carbon chemical shift. The recoupling time for HC is typically set to half the HN recoupling time to account for the approximately 2-fold difference in the coupling strength. (C) Depiction of the torsion angles, φ_H_, and the projection angles, φ_proj_. (D) Simulated MODER5-MODER5 recoupling curves with different torsion angles. For HC and NH groups, *D*_HC_ = 22 kHz and *D*_HN_ = 11 kHz. The MAS rate is 55.555 kHz. Curves from black to light gray are from 90°–120° and from violet to blue are 130°–180°. Two input bond angles are used, θ_NCαHα_ = 71° and θ_HNCα_ = 120°.

In Figure 1D, we show the torsion angle dependent variation of the MODER5-MODER5 oscillation curve in the 90°–180° angle range. The dependence is symmetric around 90°. Transformation from the torsion angle eliminates certain projection angle possibilities such that the torsion angle determination is sensitive and unique between 150° and 180° and there are two possible torsion angle values between 90° and 150° at short mixing times.^13^

Determination of HCα and HN dipolar couplings serves as the first step for obtaining precise torsion angles. To determine HCα and HN dipolar couplings, respectively, we used the same pulse sequence as in Figure 1A but evolve HCα or HN recoupling separately. In Figure 2, we show the MODER5 dipolar oscillation curves of selected residues for both U–^13^C-^15^N labeled and α-PET labeled SH3: V9 (A, B) and G51 (C, D). For each sample, we determined dipolar coupling values with precision below 500 Hz. Fits for additional resolved residues are shown in Figures S4–S9, including consideration of imperfect α-PET labeling.

**Figure 2:**
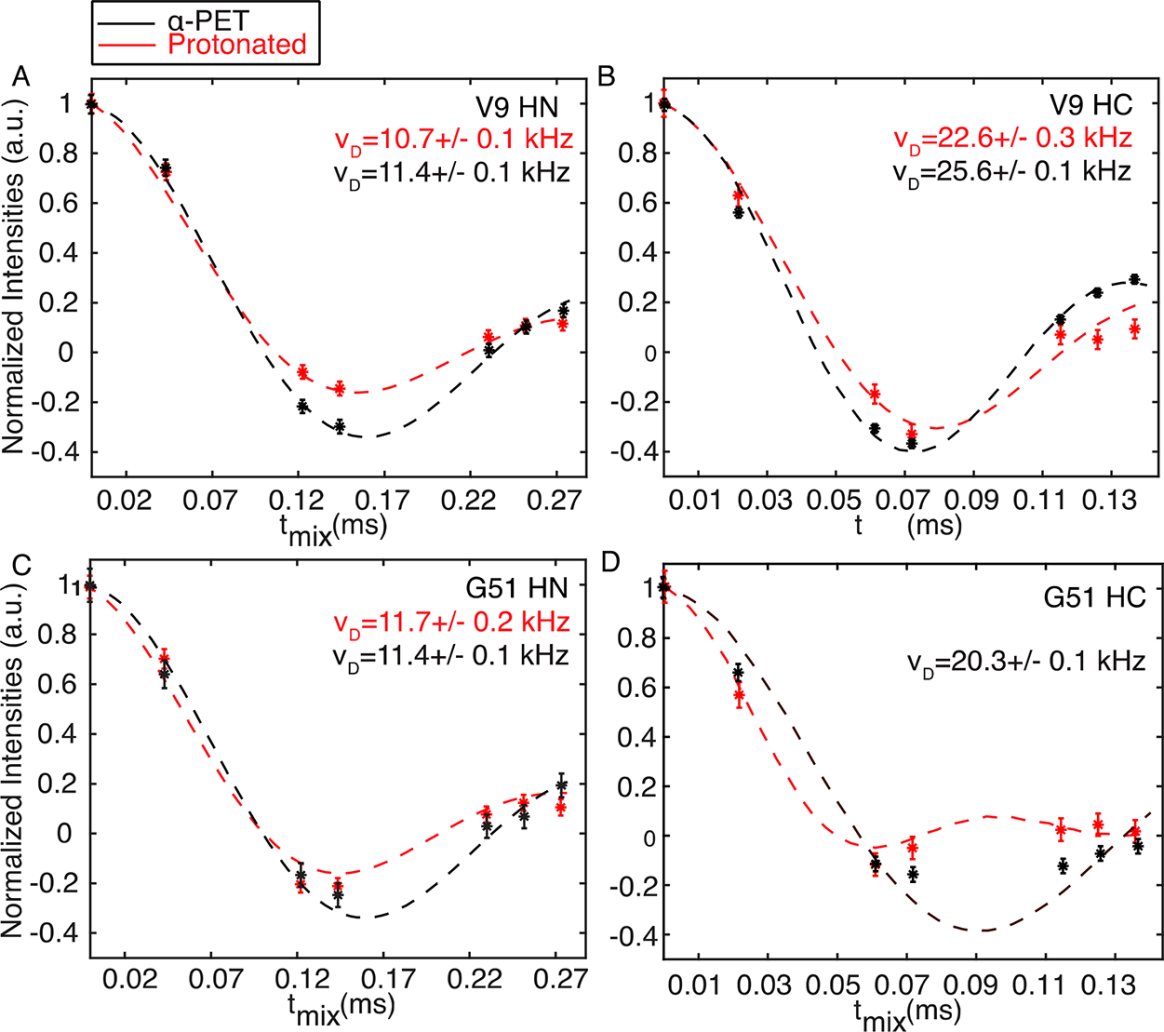
Signal amplitude modulation during MODER5 dipolar recoupling of H–N (A, C) or H–C_α_ (B, D) couplings, which are dominated by H–N and H–C_α_ spin pairs. Protonated SH3 is shown in red, and α-PET labeled SH3 is shown in black. Fit curves are shown as dashed lines. Data were recorded on a 600 MHz Bruker spectrometer and 55.555 kHz MAS and fitted with in-house MATLAB code using exact numerical simulations. Fitting in part (D) assumes two protons attached to the α carbon in the protonated case (red line). The error is estimated with 100 Monte Carlo curves from the experimental signal to noise ratio (SNR). Error bars on the points were determined from the signal-to-noise ratio of the spectra.

Figure S10 shows the simulated case of torsion angle curves for two alpha protons (Gly) and the influence of RF inhomogeneity. For the fully protonated SH3 sample, the small T_2,eff_ and the presence of two protons on Gly decreases the sensitivity to the torsion angle values. The α-PET sample is therefore preferable, in particular for glycine (Figure 2D). The downside of using this labeling scheme is that Arg residues are not efficiently labeled.^39^

A more pronounced dipolar oscillation was observed for short, 300 μs, H–C CP (Figure S4) compared with 1.5 ms CP (Figure S5). This can be explained by RF inhomogeneities of the probe and selection of signal with a more homogeneous field distribution in short CP.^43−46^ For α-PET SH3, short CP is also important since it transfers polarization primarily between directly bonded atoms and excludes any nonprotonated Cα signal that may be present due to imperfect labeling (Figure S8).

Following determination of the dipolar coupling values, the torsion angle determination is carried out using the same experimental settings, such as CP power levels and times. In Figure 3 we show the determination of φ_H_ torsion angles in α-PET SH3. Figure 3A,B shows the best fit torsion angles for residues V9 and G51 and reduced χ^2^ (χ_v_^2^) analysis. All 300 χ_v_^2^ curves from a Monte Carlo analysis (random adjustment of the points by one standard deviation) are shown in gray (Figure 3C,D). The averaged value is denoted with the black curve. As shown in Figure 3, we determined V9 φ_H_ to be 152° and G51 to be 135.2°. A comparison to the crystal structures is shown as the dotted lines in blue and black and the TALOSN prediction is marked by the dotted line in red.

**Figure 3:**
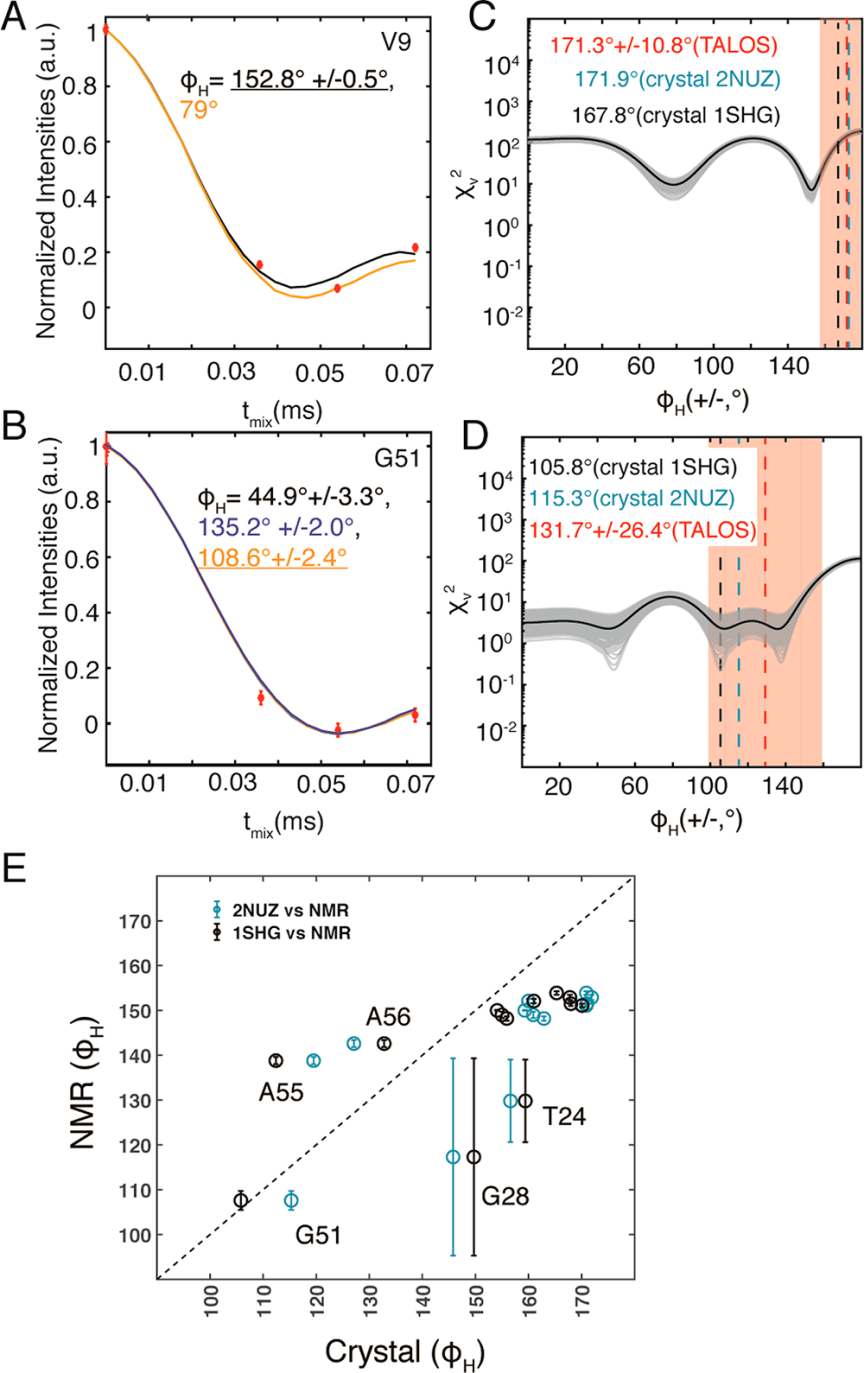
(H)(CA)NH-MODERN torsion angle determination applied to the model protein SH3. (A, B) MODERN decay curve for selected residues, V9 and G51. Best fit curves are shown as solid lines. The error reported in the fit angle was generated using the Monte Carlo method and the experimental SNR and is indicated at 1.5 times the standard deviation. (C, D) reduced χ^2^ plots for the 300 fits in the Monte Carlo analysis (experimental points were adjusted according to the spectrum noise level). The average is shown in black. Shown in red are the TALOS-N predictions with error estimates (1.5 standard deviation), shaded in orange. Torsion angle values extracted from X-ray crystal structures are shown in cyan (pdb: 2NUZ) and black (pdb: 1SHG). (E) Correlation plot of torsion angles from crystal structures (pdb: 2NUZ (cyan), 1SHG (black)) against all 13 SH3 φ_H_ angles determined from (H)(CA)NH-MODERN spectra. Error bars are shown at 1.5 times the estimated standard deviation, with the exception of G28, for which a low fit quality was evident (details in the Supporting Information). NMR data was recorded on a 600 MHz Bruker spectrometer, with 55.555 kHz MAS. Experimental torsion angle data for all residues is shown in Figures S12–S14.

Figure 3E shows a correlation plot for the φ_H_ angle determined for 13 residues by NMR and taken from the crystal structure. A clear correlation is seen, while still for some residues ∼20° differences are observed. Note that the crystal structure, even at a high resolution of 1.8 Å, contributes to some of the discrepancy. Two independently solved crystal structures at 1.0 and 1.1 Å were found to differ by about 4.7°.^47^ For SH3 at ∼1.8 Å, a similar deviation of 5° occurs for the crystal structures 2NUZ and 1SHG, among the 13 residues for which we determined the angle by NMR. Another potentially important reason for these differences is the transformation of NMR determined projection angles to torsion angles. Variation of the angles θ′_NCαHα_ and θ_HNCα_ by 4° results in ∼6° differences in torsion angle (Figure S11). While here we demonstrated angle determination for highly homogeneous preparations, it would be straightforward to extend the method to consider some sample inhomogeneity, by considering a distribution of angles. A comparison of the obtained torsion angle values for the two labeling schemes and different contact times is shown in Figure S15.

For wide applicability to biological samples, it is important that the method can separate resonances in 3 spectral dimensions. We demonstrate this pseudo-4D spectrum in Figure 4 with the fully protonated M2 protein from influenza A virus, using a construct that includes residues 18–60. This noncrystalline sample was prepared in lipid bilayers and displays less-ideal line widths as compared with microcrystalline SH3. In between the transmembrane helix and amphipathic helix, there is a tight and rigid turn at residues L46 and F47.^48^ This results in a deviation from the ∼130° φ_H_ angle in helices, to 167.8° for F47, as indicated in the oriented sample NMR structure, PDB 2L0J.^48^ H37 lies in the transmembrane helix, and indeed the MAS NMR determined torsion angle φ_H_ is 130° for H37 (Figure 4A,B) in good agreement with 126° in PDB 2L0J. The turn is detected via a 162° φ_H_ determination for F47 in excellent agreement with the oriented sample NMR data. Note that the lipid composition of the oriented sample differs from the one used here, notably in that a higher lipid-to-protein ratio was used.

**Figure 4:**
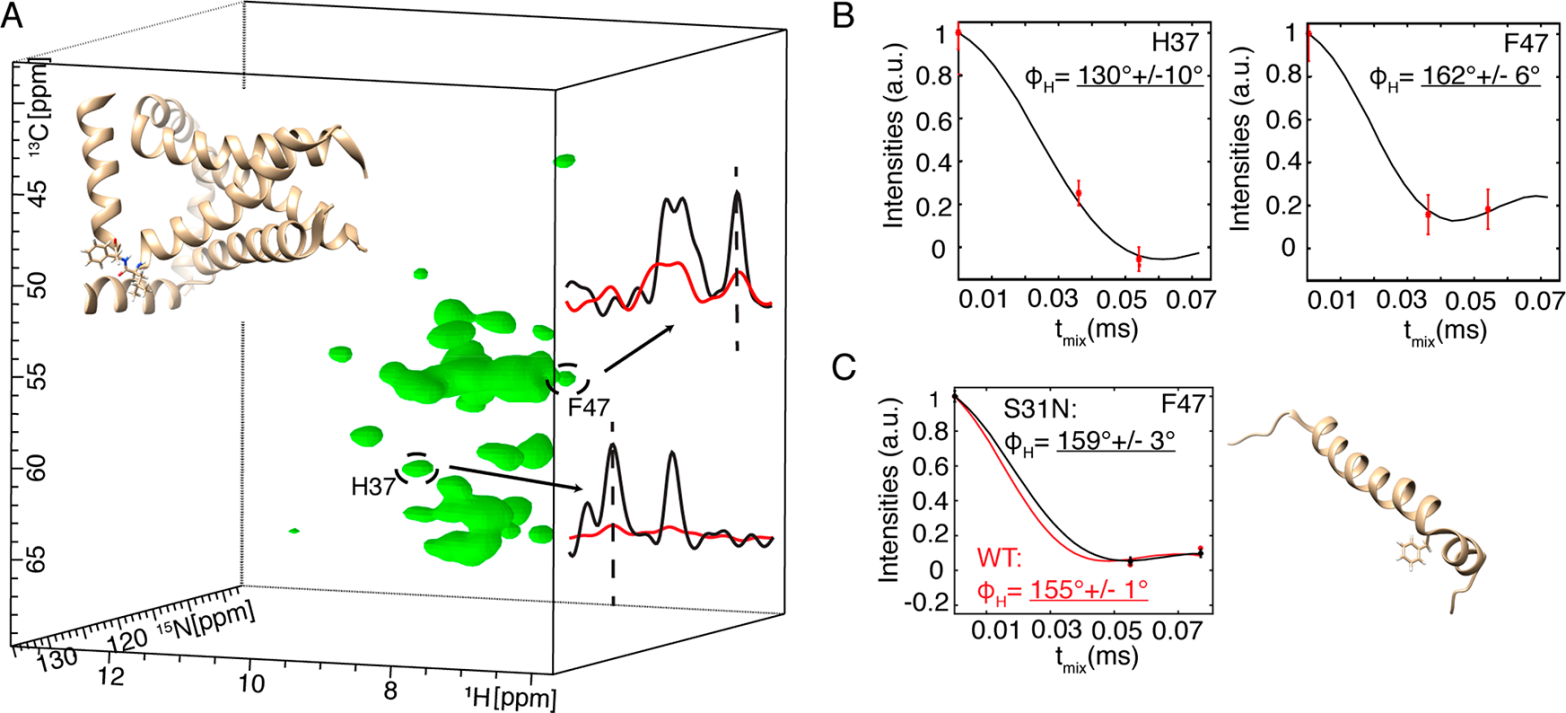
Torsion angle determination for influenza M2 protein. (A) 3D representation of the first time point of the pseudo-4D (H)CANH-MODERN experiment. The structure of M2 (PDB 2L0J) is shown as ribbons, with a turn at residues L46–F47 shown as sticks. Residues H37 and F47 are encircled, and 1D slices of the first point (black) and last point (red) in the MODERN oscillation are shown. (B) Best fit curves and torsion angle values for residues H37 and F47 from the pseudo-4D. Data of (A) and (B) are from an 800 MHz Bruker spectrometer using a 1.3 mm probe with 55.555 kHz MAS. (C) Best fit curves and torsion angle values for F47 in wild-type M2 (red) and S31N mutant (black). Measurement was performed at a 950 MHz Bruker spectrometer using a 0.7 mm probe with a MAS rate of 90.909 kHz. The data from panel (C) is pseudo-3D. The structure is from PDB 2N70 (ref (49)).

In Figure 4C, we further confirmed the F47 torsion angle using 90.909 kHz MAS with a 950 MHz instrument. This higher field and faster spinning better separate resonances, such that we chose to record pseudo-3D spectra. We also compared the S31N mutant, for which we measured a similar torsion angle, φ_H_, of 155° for F47. This value can be compared with the S31N mutant structure (PDB: 2N70) in which φ_H_ is 156°.^49^ However, 2N70 was determined from a set of distances and TALOS torsion angles, which may introduce more error in the backbone angles as compared with the direct determination of backbone angles which underlies the oriented sample structure 2L0J. Despite differences in sample preparation and measurement techniques, a consensus emerges for the F47 angle in lipid preparations. Additional experimental data for W41 and F47 is shown in Figure S16, the influence of different CP ramps is explored in Figure S17, and a set of 15 well resolved residues are shown in Figures S18–S23.

In conclusion, we demonstrated a new method for torsion angle determination at ultrafast MAS with proton detection. We measured torsion angles in both protonated and partly deuterated, α-PET-labeled SH3, which resulted in high sensitivity and led to high measurement precision, including for the previously difficult glycine residues. For the structurally important GxxxG and related motifs in membrane proteins and fibrils,^50−53^ the method is expected to provide a sensitive probe for changes in backbone secondary structure that occur for biologically significant events such as pharmaceutical binding. We also measured torsion angles for two variants of the influenza A M2 protein. M2 is more challenging in terms of signal resolution and sensitivity and proves the applicability of the method using pseudo-4D data for more challenging membrane protein samples.

## Supporting Information Available

Detailed description of dipolar coupling and phi angle determination and the MATLAB scripts used for this purpose as well as further details of sample preparation and NMR measurement conditions (PDF)
Example MATLAB files and code for NMR simulation and angle determination (ZIP)

## References

[ref1] Ramachandran G. N.; Ramakrishnan C.; Sasisekharan V. Stereochemistry of polypeptide chain configurations. J. Mol. Biol. 1963, 7 (1), 95–99. 10.1016/S0022-2836(63)80023-6. 13990617

[ref2] Dunbrack R. L.; Karplus M. Backbone-dependent Rotamer Library for Proteins Application to Side-chain Prediction. J. Mol. Biol. 1993, 230 (2), 543–574. 10.1006/jmbi.1993.1170. 8464064

[ref3] Feng X.; Verdegem P. J. E.; Lee Y. K.; Sandström D.; Edén M.; Bovee-Geurts P.; de Grip W. J.; Lugtenburg J.; de Groot H. J. M.; Levitt M. H. Direct Determination of a Molecular Torsional Angle in the Membrane Protein Rhodopsin by Solid-State NMR. J. Am. Chem. Soc. 1997, 119 (29), 6853–6857. 10.1021/ja970710d.

[ref4] Hong M.; Gross J. D.; Griffin R. G. Site-Resolved Determination of Peptide Torsion Angle φ from the Relative Orientations of Backbone N–H and C–H Bonds by Solid-State NMR. J. Phys. Chem. B 1997, 101 (30), 5869–5874. 10.1021/jp970887u.

[ref5] Karplus M.; Grant D. M. A Criterion for Orbital Hybridization and Charge Distribution in Chemical Bonds. Proc. Natl. Acad. Sci. U. S. A. 1959, 45 (8), 1269–73. 10.1073/pnas.45.8.1269. 16590503 PMC222709

[ref6] Pardi A.; Billeter M.; Wuthrich K. Calibration of the angular dependence of the amide proton-C alpha proton coupling constants, 3JHN alpha, in a globular protein. Use of 3JHN alpha for identification of helical secondary structure. J. Mol. Biol. 1984, 180 (3), 741–51. 10.1016/0022-2836(84)90035-4. 6084720

[ref7] Wagner G.; Braun W.; Havel T. F.; Schaumann T.; Go N.; Wuthrich K. Protein structures in solution by nuclear magnetic resonance and distance geometry. The polypeptide fold of the basic pancreatic trypsin inhibitor determined using two different algorithms, DISGEO and DISMAN. J. Mol. Biol. 1987, 196 (3), 611–39. 10.1016/0022-2836(87)90037-4. 2445992

[ref8] Clore G. M.; Appella E.; Yamada M.; Matsushima K.; Gronenborn A. M. Determination of the secondary structure of interleukin-8 by nuclear magnetic resonance spectroscopy. J. Biol. Chem. 1989, 264 (32), 18907–11. 10.1016/S0021-9258(19)47243-8. 2681204

[ref9] Reif B.; Hennig M.; Griesinger C. Direct Measurement of Angles Between Bond Vectors in High-Resolution NMR. Science 1997, 276 (5316), 1230–1233. 10.1126/science.276.5316.1230. 9157875

[ref10] Sabo T. M.; Gapsys V.; Walter K. F. A.; Fenwick R. B.; Becker S.; Salvatella X.; de Groot B. L.; Lee D.; Griesinger C. Utilizing dipole-dipole cross-correlated relaxation for the measurement of angles between pairs of opposing CαHα-CαHα bonds in anti-parallel β-sheets. Methods 2018, 138–139, 85–92. 10.1016/j.ymeth.2018.04.007. 29656081

[ref11] Hong M.; Gross J. D.; Hu W.; Griffin R. G. Determination of the Peptide Torsion Angle φ by15N Chemical Shift and13Cα-1HαDipolar Tensor Correlation in Solid-State MAS NMR. J. Magn. Reson. 1998, 135 (1), 169–177. 10.1006/jmre.1998.1573. 9799691

[ref12] Bower P. V.; Oyler N.; Mehta M. A.; Long J. R.; Stayton P. S.; Drobny G. P. Determination of Torsion Angles in Proteins and Peptides Using Solid State NMR. J. Am. Chem. Soc. 1999, 121 (36), 8373–8375. 10.1021/ja991330q.

[ref13] Hong M. Determination of Multiple φ-Torsion Angles in Proteins by Selective and Extensive 13C Labeling and Two-Dimensional Solid-State NMR. J. Magn. Reson. 1999, 139 (2), 389–401. 10.1006/jmre.1999.1805. 10423377

[ref14] Rienstra C. M.; Hohwy M.; Mueller L. J.; Jaroniec C. P.; Reif B.; Griffin R. G. Determination of Multiple Torsion-Angle Constraints in U–13C,15N-Labeled Peptides: 3D1H–15N–13C–1H Dipolar Chemical Shift NMR Spectroscopy in Rotating Solids. J. Am. Chem. Soc. 2002, 124 (40), 11908–11922. 10.1021/ja020802p. 12358535

[ref15] Chan J. C. C.; Tycko R. Solid-State NMR Spectroscopy Method for Determination of the Backbone Torsion Angle ψ in Peptides with Isolated Uniformly Labeled Residues. J. Am. Chem. Soc. 2003, 125 (39), 11828–11829. 10.1021/ja0369820. 14505399

[ref16] Shen Y.; Bax A. Protein backbone chemical shifts predicted from searching a database for torsion angle and sequence homology. J. Biomol. NMR 2007, 38 (4), 289–302. 10.1007/s10858-007-9166-6. 17610132

[ref17] Shen Y.; Delaglio F.; Cornilescu G.; Bax A. TALOS+: a hybrid method for predicting protein backbone torsion angles from NMR chemical shifts. J. Biomol. NMR 2009, 44 (4), 213–223. 10.1007/s10858-009-9333-z. 19548092 PMC2726990

[ref18] Shen Y.; Bax A. SPARTA+: a modest improvement in empirical NMR chemical shift prediction by means of an artificial neural network. J. Biomol. NMR 2010, 48 (1), 13–22. 10.1007/s10858-010-9433-9. 20628786 PMC2935510

[ref19] Shen Y.; Roche J.; Grishaev A.; Bax A. Prediction of nearest neighbor effects on backbone torsion angles and NMR scalar coupling constants in disordered proteins. Protein Sci. 2018, 27 (1), 146–158. 10.1002/pro.3292. 28884933 PMC5734315

[ref20] Shen Y.; Bax A. Protein backbone and sidechain torsion angles predicted from NMR chemical shifts using artificial neural networks. J. Biomol. NMR 2013, 56 (3), 227–241. 10.1007/s10858-013-9741-y. 23728592 PMC3701756

[ref21] Chevelkov V.; Rehbein K.; Diehl A.; Reif B. Ultrahigh resolution in proton solid-state NMR spectroscopy at high levels of deuteration. Angew. Chem., Int. Ed. 2006, 45 (23), 3878–81. 10.1002/anie.200600328. 16646097

[ref22] Agarwal V.; Penzel S.; Szekely K.; Cadalbert R.; Testori E.; Oss A.; Past J.; Samoson A.; Ernst M.; Bockmann A.; Meier B. H. De novo 3D structure determination from sub-milligram protein samples by solid-state 100 kHz MAS NMR spectroscopy. Angew. Chem., Int. Ed. 2014, 53 (45), 12253–6. 10.1002/anie.201405730. 25225004

[ref23] Andreas L. B.; Le Marchand T.; Jaudzems K.; Pintacuda G. High-resolution proton-detected NMR of proteins at very fast MAS. J. Magn. Reson. 2015, 253, 36–49. 10.1016/j.jmr.2015.01.003. 25797003

[ref24] Barbet-Massin E.; Huang C.-T.; Daebel V.; Hsu S.-T. D.; Reif B. Site-Specific Solid-State NMR Studies of “Trigger Factor” in Complex with the Large Ribosomal Subunit50S. Angew. Chem., Int. Ed. 2015, 54 (14), 4367–4369. 10.1002/anie.201409393. 25655173

[ref25] Andreas L. B.; Jaudzems K.; Stanek J.; Lalli D.; Bertarello A.; Le Marchand T.; Cala-De Paepe D.; Kotelovica S.; Akopjana I.; Knott B.; Wegner S.; Engelke F.; Lesage A.; Emsley L.; Tars K.; Herrmann T.; Pintacuda G. Structure of fully protonated proteins by proton-detected magic-angle spinning NMR. Proc. Natl. Acad. Sci. U. S. A. 2016, 113 (33), 9187–92. 10.1073/pnas.1602248113. 27489348 PMC4995937

[ref26] Stanek J.; Andreas L. B.; Jaudzems K.; Cala D.; Lalli D.; Bertarello A.; Schubeis T.; Akopjana I.; Kotelovica S.; Tars K.; Pica A.; Leone S.; Picone D.; Xu Z. Q.; Dixon N. E.; Martinez D.; Berbon M.; El Mammeri N.; Noubhani A.; Saupe S.; Habenstein B.; Loquet A.; Pintacuda G. NMR Spectroscopic Assignment of Backbone and Side-Chain Protons in Fully Protonated Proteins: Microcrystals, Sedimented Assemblies, and Amyloid Fibrils. Angew. Chem., Int. Ed. 2016, 55 (50), 15503–15509. 10.1002/anie.201685061. 27865050

[ref27] Linser R.; Fink U.; Reif B. Proton-detected scalar coupling based assignment strategies in MAS solid-state NMR spectroscopy applied to perdeuterated proteins. J. Magn. Reson. 2008, 193 (1), 89–93. 10.1016/j.jmr.2008.04.021. 18462963

[ref28] Fricke P.; Chevelkov V.; Zinke M.; Giller K.; Becker S.; Lange A. Backbone assignment of perdeuterated proteins by solid-state NMR using proton detection and ultrafast magic-angle spinning. Nat. Protoc. 2017, 12 (4), 764–782. 10.1038/nprot.2016.190. 28277547

[ref29] Chevelkov V.; Fink U.; Reif B. Accurate Determination of Order Parameters from 1H,15N Dipolar Couplings in MAS Solid-State NMR Experiments. J. Am. Chem. Soc. 2009, 131 (39), 14018–14022. 10.1021/ja902649u. 19743845

[ref30] Schanda P.; Meier B. H.; Ernst M. Quantitative Analysis of Protein Backbone Dynamics in Microcrystalline Ubiquitin by Solid-State NMR Spectroscopy. J. Am. Chem. Soc. 2010, 132 (45), 15957–15967. 10.1021/ja100726a. 20977205

[ref31] Hou G.; Lu X.; Vega A. J.; Polenova T. Accurate measurement of heteronuclear dipolar couplings by phase-alternating R-symmetry (PARS) sequences in magic angle spinning NMR spectroscopy. J. Chem. Phys. 2014, 141 (10), 104202 10.1063/1.4894226. 25217909 PMC4162908

[ref32] Xue K.; Mamone S.; Koch B.; Sarkar R.; Reif B. Determination of methyl order parameters using solid state NMR under off magic angle spinning. J. Biomol. NMR 2019, 73 (8–9), 471–475. 10.1007/s10858-019-00253-5. 31407204

[ref33] Xue K.; Mühlbauer M.; Mamone S.; Sarkar R.; Reif B. Accurate Determination of 1H-15N Dipolar Couplings Using Inaccurate Settings of the Magic Angle in Solid-State NMR Spectroscopy. Angew. Chem., Int. Ed. 2019, 58 (13), 4286–4290. 10.1002/anie.201814314. 30694593

[ref34] Huber M.; Hiller S.; Schanda P.; Ernst M.; Bockmann A.; Verel R.; Meier B. H. A proton-detected 4D solid-state NMR experiment for protein structure determination. ChemPhysChem 2011, 12 (5), 915–8. 10.1002/cphc.201100062. 21442705

[ref35] Najbauer E. E.; Movellan K. T.; Schubeis T.; Schwarzer T.; Castiglione K.; Giller K.; Pintacuda G.; Becker S.; Andreas L. B. Probing Membrane Protein Insertion into Lipid Bilayers by Solid-State NMR. ChemPhysChem 2018, 302–310. 10.1002/cphc.201800793. 30452110

[ref36] Zhang X. C.; Forster M. C.; Nimerovsky E.; Movellan K. T.; Andreas L. B. Transferred-Rotational-Echo Double Resonance. J. Phys. Chem. A 2021, 125 (3), 754–769. 10.1021/acs.jpca.0c09033. 33464081 PMC7884007

[ref37] Nishiyama Y.; Zhang R.; Ramamoorthy A. Finite-pulse radio frequency driven recoupling with phase cycling for 2D 1H/1H correlation at ultrafast MAS frequencies. J. Magn. Reson. 2014, 243, 25–32. 10.1016/j.jmr.2014.03.004. 24713171 PMC4037380

[ref38] Zhang R.; Mroue K. H.; Ramamoorthy A. Proton-Based Ultrafast Magic Angle Spinning Solid-State NMR Spectroscopy. Acc. Chem. Res. 2017, 50 (4), 1105–1113. 10.1021/acs.accounts.7b00082. 28353338 PMC5828698

[ref39] Movellan K. T.; Najbauer E. E.; Pratihar S.; Salvi M.; Giller K.; Becker S.; Andreas L. B. Alpha protons as NMR probes in deuterated proteins. J. Biomol. NMR 2019, 73 (1), 81–91. 10.1007/s10858-019-00230-y. 30762170 PMC6441447

[ref40] Nimerovsky E.; Soutar C. P. A modification of γ-encoded RN symmetry pulses for increasing the scaling factor and more accurate measurements of the strong heteronuclear dipolar couplings. J. Magn. Reson. 2020, 319, 106827 10.1016/j.jmr.2020.106827. 32950918

[ref41] Levitt M. H. Symmetry in the design of NMR multiple-pulse sequences. J. Chem. Phys. 2008, 128 (5), 052205 10.1063/1.2831927. 18266410

[ref42] Pines A.; Gibby M. G.; Waugh J. S. Proton-Enhanced Nmr of Dilute Spins in Solids. J. Chem. Phys. 1973, 59 (2), 569–590. 10.1063/1.1680061.

[ref54] Paulson E. K.; Martin R. W.; Zilm K. W. Cross Polarization, Radio Frequency Field Homogeneity, and Circuit Balancing in High Field Solid State NMR Probes. J. Magn. Reson. 2004, 171 (2), 314–323. 10.1016/j.jmr.2004.09.009. 15546758

[ref55] Engelke F. Electromagnetic Wave Compression and Radio Frequency Homogeneity in NMR Solenoidal Coils: Computational Approach. Concepts Magn. Reson. 2002, 15 (2), 129–155. 10.1002/cmr.10029.

[ref56] Gupta R.; Hou G.; Polenova T.; Vega A. J. RF Inhomogeneity and how it controls CPMAS. Solid State Nucl. Magn. Reson. 2015, 72, 17–26. 10.1016/j.ssnmr.2015.09.005. 26422256 PMC4674349

[ref43] Tošner Z.; Purea A.; Struppe J. O.; Wegner S.; Engelke F.; Glaser S. J.; Reif B. Radiofrequency fields in MAS solid state NMR probes. J. Magn. Reson. 2017, 284, 20–32. 10.1016/j.jmr.2017.09.002. 28946058

[ref44] Tošner Z.; Sarkar R.; Becker-Baldus J.; Glaubitz C.; Wegner S.; Engelke F.; Glaser S. J.; Reif B. Overcoming Volume Selectivity of Dipolar Recoupling in Biological Solid-State NMR Spectroscopy. Angew. Chem., Int. Ed. 2018, 57 (44), 14514–14518. 10.1002/anie.201805002. 29989288

[ref45] Nishimura K.; Fu R.; Cross T. A. The Effect of RF Inhomogeneity on Heteronuclear Dipolar Recoupling in Solid State NMR: Practical Performance of SFAM and REDOR. J. Magn. Reson. 2001, 152 (2), 227–233. 10.1006/jmre.2001.2410.

[ref46] Gupta R.; Hou G.; Polenova T.; Vega A. J. RF inhomogeneity and how it controls CPMAS. Solid State Nucl. Magn. Reson. 2015, 72, 17–26. 10.1016/j.ssnmr.2015.09.005. 26422256 PMC4674349

[ref47] Wang A. C.; Bax A. Determination of the Backbone Dihedral Angles φ in Human Ubiquitin from Reparametrized Empirical Karplus Equations. J. Am. Chem. Soc. 1996, 118 (10), 2483–2494. 10.1021/ja9535524.

[ref48] Sharma M.; Yi M.; Dong H.; Qin H.; Peterson E.; Busath D. D.; Zhou H. X.; Cross T. A. Insight into the Mechanism of the Influenza A Proton Channel from a Structure in a Lipid Bilayer. Science 2010, 330 (6003), 509–512. 10.1126/science.1191750. 20966252 PMC3384994

[ref49] Andreas L. B.; Reese M.; Eddy M. T.; Gelev V.; Ni Q. Z.; Miller E. A.; Emsley L.; Pintacuda G.; Chou J. J.; Griffin R. G. Structure and Mechanism of the Influenza A M218–60 Dimer of Dimers. J. Am. Chem. Soc. 2015, 137 (47), 14877–14886. 10.1021/jacs.5b04802. 26218479 PMC4943461

[ref50] Senes A.; Engel D. E.; DeGrado W. F. Folding of helical membrane proteins: the role of polar, GxxxG-like and proline motifs. Curr. Opin. Struct. Biol. 2004, 14 (4), 465–479. 10.1016/j.sbi.2004.07.007. 15313242

[ref51] Faingold O.; Cohen T.; Shai Y. A GxxxG-like Motif within HIV-1 Fusion Peptide Is Critical to Its Immunosuppressant Activity, Structure, and Interaction with the Transmembrane Domain of the T-cell Receptor. J. Biol. Chem. 2012, 287 (40), 33503–33511. 10.1074/jbc.M112.370817. 22872636 PMC3460451

[ref52] Decock M.; Stanga S.; Octave J.-N.; Dewachter I.; Smith S. O.; Constantinescu S. N.; Kienlen-Campard P. Glycines from the APP GXXXG/GXXXA Transmembrane Motifs Promote Formation of Pathogenic Aβ Oligomers in Cells. Front. Aging Neurosci. 2016, 8, 107 10.3389/fnagi.2016.00107. 27242518 PMC4861705

[ref53] Sarkar D.; Chakraborty I.; Condorelli M.; Ghosh B.; Mass T.; Weingarth M.; Mandal A. K.; La Rosa C.; Subramanian V.; Bhunia A. Self-Assembly and Neurotoxicity of β-Amyloid (21–40) Peptide Fragment: The Regulatory Role of GxxxG Motifs. ChemMedChem 2020, 15 (3), 293–301. 10.1002/cmdc.201900620. 31762186

