# Supplementary material for: Backbone Torsion Angle Determination Using Proton Detected Magic-Angle Spinning Nuclear Magnetic Resonance
Source: J Phys Chem Lett. 2021 Dec 27;13(1):18–24. doi: 10.1021/acs.jpclett.1c03267 (PMC8762656; doi:10.1021/acs.jpclett.1c03267)
Supplement: Supplementary file 1 — jz1c03267_si_001.pdf [file jz1c03267_si_001.pdf]

Backbone Torsion Angle Determination Using Proton Detected Magic-Angle Spinning Nuclear Magnetic Resonance

Kai Xue,[+a*] Evgeny Nimerovsky,[+a] Kumar A. Tekwani Movellan,[a,#] Stefan Becker,[a] Loren B. Andreas[a *]

[a] Max Planck Institute for Biophysical Chemistry, Department of NMR Based Structural Biology, Am Fassberg. 11, Goettingen, Germany

[#] Current Address: Department of Chemistry and Biochemistry, University of Delaware, Newark, DE 19716, United States

To whom correspondence should be addressed: kaxu@nmr.mpipc.mpg.de land@nmr.mpipc.mpg.de

[+]KX and EN contribute equally to this work

Table of Contents

Dipolar Coupling Determination **2**

Torsion Angle Determination **14**

M2 data, 950 MHz spectrometer **23**

MATLAB Code **29**

Sample preparation **35**

NMR Measurements **35**

Dipolar Coupling Determination

MODER5 consists of two 90° pulses with phases 36° and 164.2° as described in Ref. [1]. In an ideal case, the frequency of the MODER5 oscillations depends only on the dipolar coupling value, ν_D. To obtain dipolar values we compare the normalized experimental MODERN signal, $\bar{S}_{exp}(t_{exp}) = S_{exp}(t_{mix})/S_{exp}(0)$ with the normalized simulated, $\bar{S}_{sim}(\nu_D; t_{mix}) = S_{sim}(\nu_D; t_{mix})/S_{sim}(0)$ and minimize the value of the residual, χ_ν^2:

$$\chi_\nu^2 = \left[sum\left[\left(\bar{S}_{exp}(t_{mix}) - \bar{S}_{sim}(\nu_D; t_{mix})\right)/error\right]^2\right]/(\nu - 1), \qquad \text{Eqn. (S1)}$$

where, ν is the number of the experimental points; *error* – is the uncertainty in the peak amplitude, estimated from the RMSD of a noise region of the spectrum (the same region for all spectra in one series). Peak intensities were analyzed using software PINT (Ref. [2])

The MODERN signal is influenced by experimental imperfections, for example, rf-field inhomogeneity. Obtaining reasonable fits required modeling a distribution of flip angles, α_{rf}, for the MODERN pulses across the coil: $\alpha_{rf} \in\leq 90^o$. This flip angle distribution primarily affects the depth of dipolar oscillations (Ref. [1], Figs. 4a and 6b). Such behavior is different from traditional γ-encoded RN pulses[3,4], in which the scaling factor is affected, meaning the apparent dipolar oscillation frequency changes. (Ref.[1], Figs. 4a and 6a).

Figure S1A demonstrates the amplitude of the first minima of the MODER5 curve (the colormap) as functions of the pulse flip angle (axis X) and the input dipolar coupling values (axis Y). Under ideal conditions (zero offset and 90° the flip angle), the amplitude of the first minima reaches -0.41 (dark blue). With increasing flip angle deviations from 90°, the performance of

MODERN pulses deteriorates, and the amplitude of the first minima is shifted to positive values (red).

The sensitivity to the flip angle deviation is increased with decreasing dipolar coupling constant. For example, Figure S1B shows the MODER5 curves with 90^0 flip angle (solid lines) and 85° flip angle (dashed lines) with strong (23 kHz, black lines) and weak (2.3 kHz, red lines) dipolar coupling values. With a 23 kHz dipolar coupling, MODER5 curves reach negative values for both cases. With a 2.3 kHz coupling, MODERN curves coincide up to ~0.1 ms. From that time the solid red curve continues to dephase (eventually reaching -0.41), whereas the dashed red line returns toward 1.

The stability of the fitting procedure with respect to RF power can be assessed by first simulating MODER5 signal under different flip angle values and input dipolar coupling values ($D_{IS,intput}$), and then fitting the simulated data (assuming perfect a flip angle) to obtain an output dipolar coupling value ($D_{IS,output}$). Again, the ideal MODER5 signal is obtained for a 90° flip angel (α_{rf}=90°), The colormap in Figure S1C demonstrates the ratio between these two values, $D_{IS,output}$/ $D_{IS,intput}$. In the ideal case this ratio is 1 (cyan). Above $D_{IS,intput}$ of 11 kHz, the output dipolar coupling is larger, while below the fit deviates towards smaller values. The deviations are symmetric with respect to the optimal value of α_{rf} as was observed for the dependence of the MODER5 amplitude (Fig S1A). As expected from Fig S1C, stronger dipole couplings result in a broader region of correct $D_{IS,output}$ values.

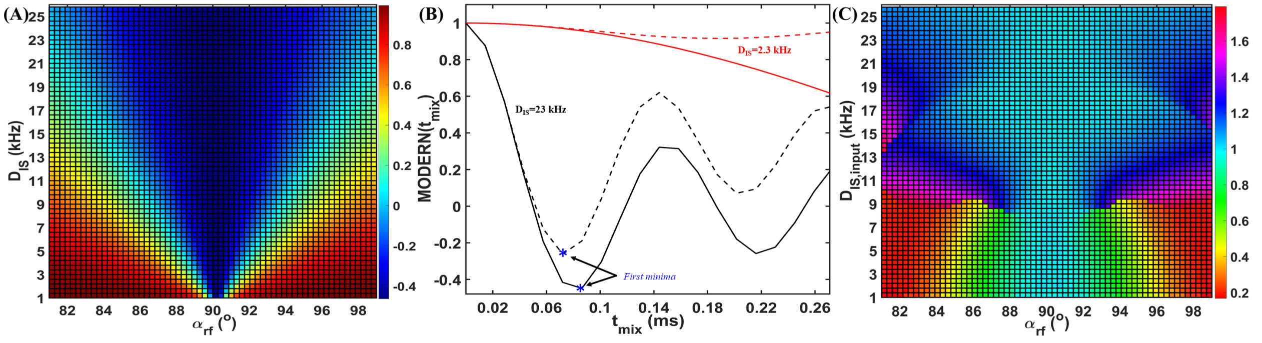

Figure S1. Simulated dependence of MODER5 pulses on flip angle deviations. (A) The value of the first minimum (marked with a star in B) as a function of a flip angle (α_{rf}, axis x) and the heteronuclear dipolar coupling values (D_{IS}, axis y). The MAS rate is 55.555 kHz. (B) The simulated MODER5 curves with strong (23.3 kHz, black lines) and weak (2.3 kHz, red lines) dipolar coupling values with ideal flip angle (90°, solid lines) and (85°, red lines). (C) The colormap shows the ratio $D_{IS,output}$/$D_{IS,input}$ as a function of α_{rf} (the flip angle) and $D_{IS,input}$ (the input dipolar coupling value). For each pixel the simulated MODER5(α_{rf},$D_{IS,input}$) was simulated and compared with MODER5(α_{rf}=90°,$D_{IS,output}$) using RMSD to obtain $D_{IS,output}$. In the ideal case $D_{IS,output}$/$D_{IS,input}$=1. In all cases the simulations were performed up to 0.14 ms

Although such sensitivity to the flip angle deviation (and rf-field inhomogeneity) impedes the measurements of the weak dipolar couplings, it can be taken into account into simulations in order to accurately measure stronger couplings, as shown below.

To confirm the reliability and the preciseness of the MODERN experiments for dipolar and torsion angle determinations, we investigated two samples: α-PET SH3 and fully protonated SH3 samples. α-PET SH3 sample was investigated twice: with short and long cross polarization (CP) transfer times from protons to carbons. For distinction, the datasets with a short and long CP are mentioned here as: α-PET SH3 (short CP) and α-PET SH3 (long CP).

To obtain the torsion angle values, the HC and NH dipolar coupling values first need to be determined. For the determination of the dipolar coupling values, MODERN pulses are applied on the proton channel while the coherence is on carbon spins (HC coupling), or nitrogen spins (HN coupling).

For each sample, **the first step in the experimental protocol** was a careful optimization of the MODER5 power level by maximizing the signal dephasing at short times. For example, at ~0.07 ms a series of 1D spectra can be recorded as function of the rf-field power. The optimal rf-field power is determinate when the MODERN signal has the lowest value. Figure S2A demonstrates the experimental HC MODER5 curve at 72 μs (expected first MODERN minimum for a 23 kHz dipolar coupling) as a function of the proton rf-field power in Watts. The applied optimal rf-field powers on HC and NH dipolar interactions were optimized separately. In our case, the applied proton rf-field powers to recouple HC and dipolar interactions had the same values of ~133 kHz (with respect to the 90° pulse calibration) for the 599 MHz magnet; 128.5 kHz and 129 kHz for the 800 MHz magnet and 218 kHz and 222 kHz for the 950 MHz magnet.

In addition to the influence of the rf-field inhomogeneity, we also found that the values of the proton to carbon CP times affected the performance of MODERN. Figure S3B demonstrates 1D HC MODER5 signal with a 72 μs MODER5 mixing time as a function of the CP time. Short mixing of 0.3 ms resulted in better performance than longer times.

After optimization of CP and rf-field powers, these same settings are used for dipolar coupling and torsion angle determination using MODERN.

For obtaining the dipolar coupling and torsion angle values, the experimental signals are compared with simulation in MATLAB (details in 'The MATLAB Code' section). Since the signal evolution is sensitive to the precise B_1 rf-field, the influence of the rf-field inhomogeneity in the

probes should be simulated. The profile of rf-field inhomogeneity in MAS probes was investigated before[5–7]. In these works, it was found that balanced probes result in symmetric profiles that are describe well by a Gaussian function. Since MODERN pulses have a symmetry around the optimal value (Fig S1), we used a half Gaussian function in simulations.

We found that reliable fits required consideration of rf inhomogeneity, which was modeled as a Gaussian flip angle distribution between 1 and a minimal value (maximum deviation, $\Delta\alpha_{rf,max}$:

$$\Delta\alpha_{rf,max} = 1 - \frac{\alpha_{rf,min}}{90^{o}} \quad \text{Eqn. (S2)}$$

To confirm the Gaussian profile of the rf-field inhomogeneity in the probe we recorded a series of 1D HCαNH experiments as a function of the length of the first proton pulse from 1.8 to 184.2 µs with a 1.6 µs step. Figure S2C shows the FFT of such signal (black stars), the Gaussian function (red line) and half of the flip angle distribution function, which is used in simulations (cyan dashed line). All experimental and simulated FFT spectra coincide very well and symmetry around maximal value is observed.

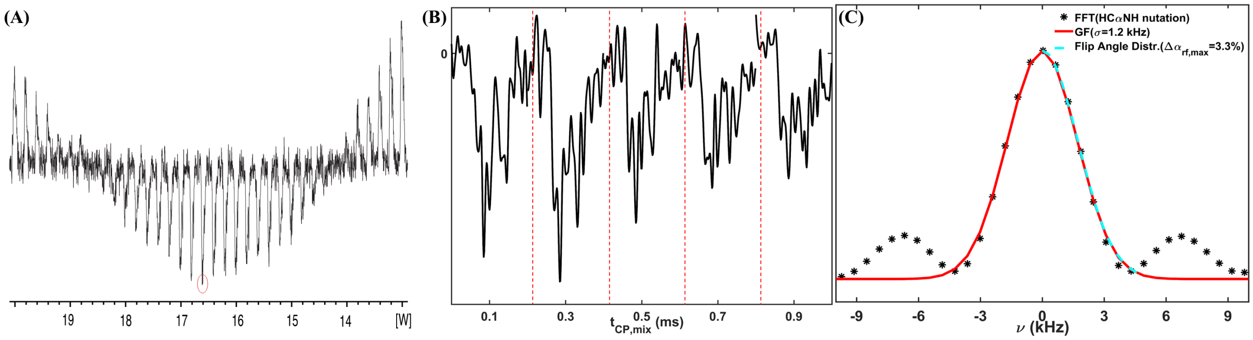

Figure S2. MODER5 optimization. (A) A series of 1D α-PET SH3 spectra obtained as a function of proton rf power (HC MODER5 pulses) measured in Watts. The recoupling time was set to 72 µs, which is the expected negative maximum for a 23 kHz dipolar coupling. The red circled point was taken as the optimal power level for 137 kHz proton recoupling. (B) MODER5 HC α-PET SH3 signal (with 72 µs of MODER5 and optimized power level) as a function of the CP time (the CP transfer from proton to carbon before MODER5). (C) Fast Fourier Transform (FFT) of 1D (HCAN)H signal as a function of the length of first proton pulse (black stars) and comparison with a Gaussian function (red solid line) and a flip angle distribution function (cyan dashed line). The sample was the influenza A M2 protein. For FFT, MATLAB software was used. The experiments were performed at 55.555 kHz MAS.

For a specific dipolar coupling value we simulate 7-14 curves with different values of the flip angle deviations $\Delta\alpha_{rf,i} \in [0{:}\Delta\alpha_{rf,max}]$. In addition, we fit an effective exponential relaxation time, $T_{2,eff}$. The total simulated MODERN signal is defined as follows:

$$\bar{S}_{sim}(\nu_D; t_{mix}) = \sum \bar{P}(\Delta\alpha_{rf,i})\, S_{sim}(\nu_D; \Delta\alpha_{rf,i}; t_{mix})\, e^{\{-t_{mix}/T_{2,eff}\}}, \quad \text{Eqn. (S3)}$$

where $T_{2,eff}$ is an effective relaxation time and $\bar{P}(\Delta\alpha_{rf,i})$ is the normalized Gaussian weight factor for $\Delta\alpha_{rf,i}$ value.

To obtain these two values ($T_{2,eff}$, $\Delta\alpha_{rf,max}$) several MODERN dipolar curves should be prepared as an input file (described in 'The MATLAB Code' section). The best MODERN curves for determination of these parameters are the curves where the oscillations with the largest amplitudes are observed and the longest MODERN mixing time is applied. For example, in our experiments the MODERN mixing time to recouple NH dipolar interactions was two times longer than for recoupling HC dipolar interactions. Therefore, we chose at least three NH MODER5 curves for each sample. Table S1 summarizes the obtained parameters. For α-PET SH3 samples (short and long CP) $T_{2,eff}$ has a smaller influence than for fully protonated SH3. The α-PET SH3 (short CP) dataset had the smallest apparent value of $\Delta\alpha_{rf,max}$, suggesting that the CP time may influence the special distribution of the detected signal e.g. the edges versus the middle of the coil.

Table S1 The obtained $\Delta\alpha_{rf,max}$ and $T_{2,eff}$ from comparison of four experimental NH MODERN curves (for each sample) with simulated curves. The obtained values are average values obtained from four residues for α-PET SH3 (short CP) dataset and three residues for the other two samples.

sample	α-PET SH3 (short CP)	α-PET SH3 (long CP)	SH3 (long CP)
$\Delta\alpha_{rf,max}$	0.028	0.093±0.037	0.078±0.01
$T_{2,eff}$ (ms)	0.648±0.23	0.72±0.27	0.257±0.014

Figure S3 shows the experimental (V9:NH, red stars) and simulated MODERN curves (lines). The dashed lines show an ideal experiment, with no experimental imperfection, such that $\Delta\alpha_{rf,i}$=0 and $T_{2,eff} \gg t_{exp}$. The data clearly do not fit this case, but fit well when non-ideal values of $\Delta\alpha_{rf,max}$ and $T_{2,eff}$ are considered (solid line).

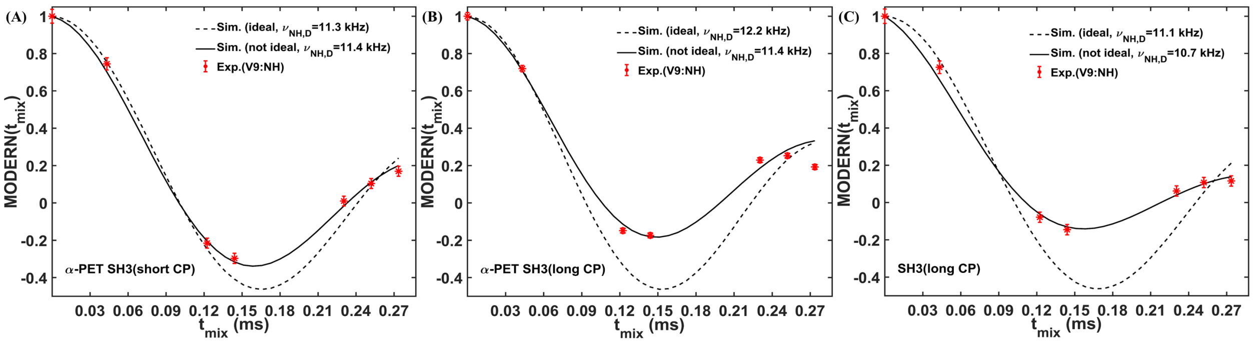

Figure S3. Experimental MODERN curves (V9:NH, red stars) and simulated curves with ideal (dashed lines) and non-ideal (solid lines) fit parameters. (A) α-PET SH3 with short CP. For non-ideal MODERN curves, $\Delta\alpha_{rf,max} = 0.028$ and

$T_{2,eff} = 0.648\, ms$. (B) α-PET SH3 with long CP mixing time. For non-ideal MODERN curves, $\Delta\alpha_{rf,max} = 0.093$ and $T_{2,eff} = 0.721\, ms$. (C) SH3 with long CP mixing time. For non-ideal MODERN curves: $\Delta\alpha_{rf,max} = 0.078$ and $T_{2,eff} = 0.257\, ms$. Measurements are performed a 55 kHz MAS. The solid lines represent the best simulated MODER5 curves with the smallest RMSD values.

Using the obtained $\Delta\alpha_{rf,max}$ and $T_{2,eff}$ from Table S1, we obtained HC and NH dipolar coupling values for α-PET SH3 (short CP and long CP) and SH3 (long CP). Figures S4-S5 show the experimental and simulated MODER5 curves for α-PET SH3 (short CP) and SH3 (long CP).

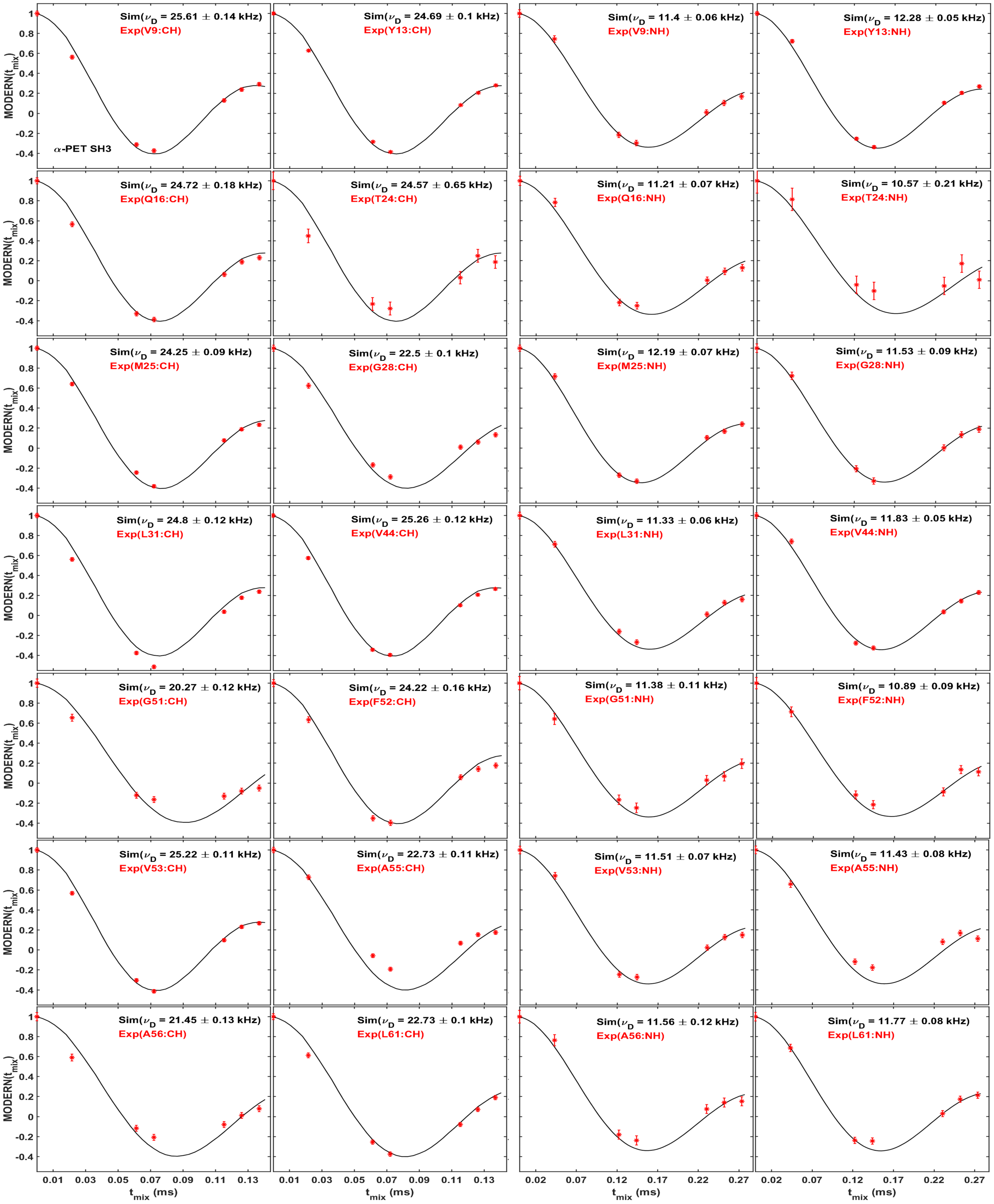

Figure S4. Experimental α-PET SH3 (short CP) and simulated MODER5 curves for HC (left two columns) and NH (right two columns) dipolar coupling values. The simulated MODER5 curves were obtained with the $\Delta\alpha_{rf,max}$ and $T_{2,eff}$ values of Table S1). Eqn. S3 represents the simulated signal. The fitting errors were obtained by generating 100 Monte Carlo curves, assuming a Gaussian noise distribution (~1.5σ).

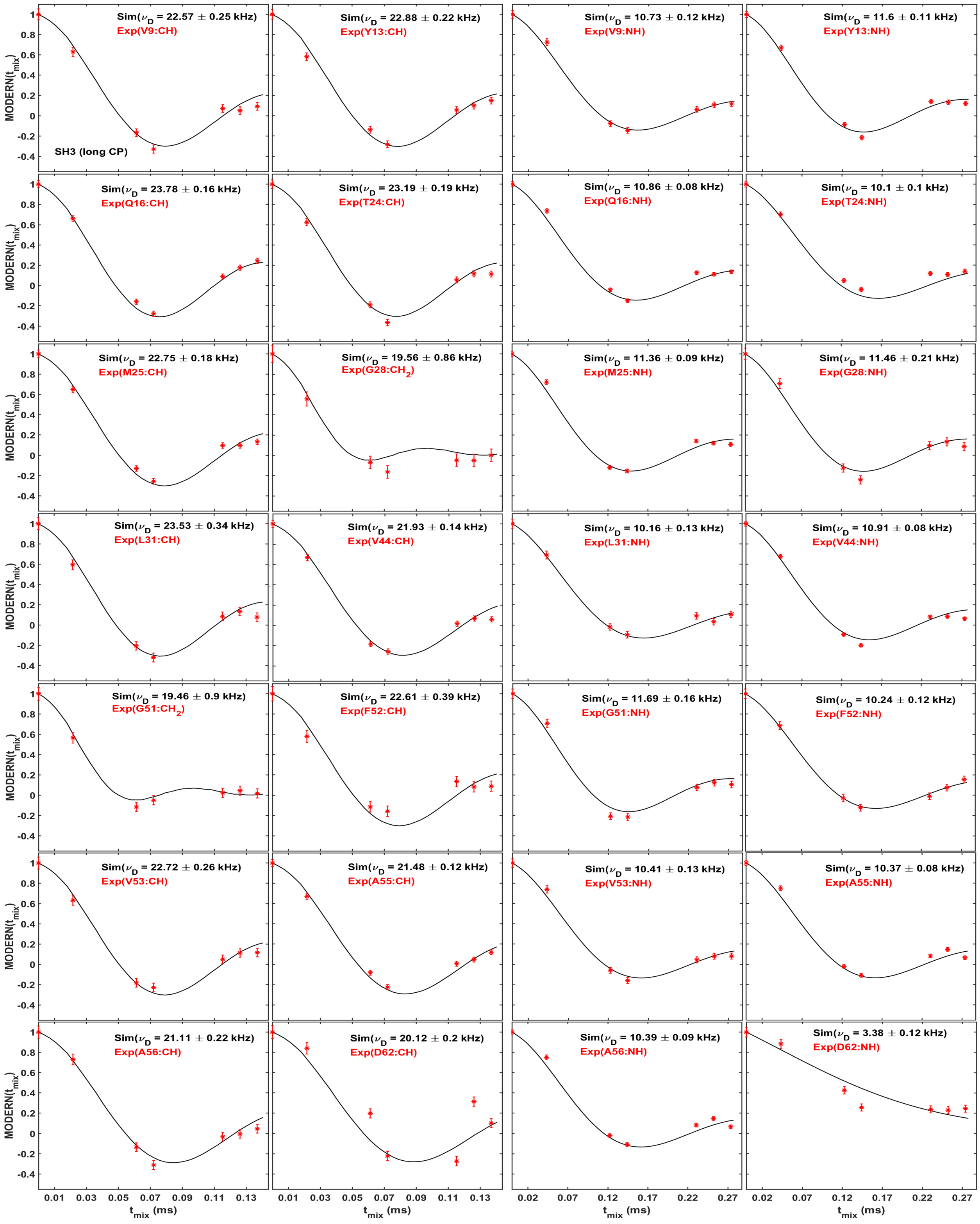

Figure S5. Experimental SH3 (long CP) and simulated MODER5 curves for HC (left two columns) and NH (right two columns) dipolar coupling values. The simulated MODER5 curves were obtained with the $\Delta\alpha_{rf,max}$ and $T_{2,eff}$ values

of Table S1). Eqn. S3 represents the simulated signal. The fitting errors were obtained by generating 100 Monte Carlo curves, assuming a Gaussian noise distribution (~1.5σ).

For processing the α-PET SH3 (long CP) MODERN data, we had to add an additional parameter, p, which defined the population of protonated C_α signal (Figure S6A). The particular α-PET sample used had incomplete incorporation of alpha protons (due to incomplete production of keto acids in the enzymatic digestion step) The deuterated alpha carbons, $D_\alpha C_\alpha$, are increasingly polarized at longer CP times, e.g. from nearby protons such as the backbone amide proton. The signal from this part of the sample is then transferred on through CP to nitrogen and then detected at the amide proton. However, there can be no CH MODERN oscillation for this part of the sample. The dipolar interaction between C_α and remote protons is ~8 times weaker in comparison to directly bonded protons. At short CP mixing times, only directly bonded HC groups are excited. However, at long mixing times, the total CP signal is the sum of the strong (directly bonded protons) and weak (remote protons) CH groups. Note that p is not a labelling population, but also depends on the local geometry of nearby protons, which explains the different p values fit to different residues of the same type (Figure S8).

For α-PET SH3 (long CP) Eqn. (3) is then rewritten as follow:

$$\bar{S}_{sim}(\nu_D; t_{mix}) =$$

$$\sum \bar{P}(\Delta\alpha_{rf,i}) \left[p \cdot S_{sim}(\nu_D; \Delta\alpha_{rf,i}; t_{mix}) + (1-p) \cdot S_{sim}(\nu_D/8; \Delta\alpha_{rf,i}; t_{mix})\right] e^{\{-t_{mix}/T_{2,eff}\}}. \quad \text{Eqn. (S4)}$$

Figure S6B shows the experimental MODER5 V9 HC curve (red stars) and simulations (solid lines), assuming full (black) and partial (red) protonation of the backbone C_α carbons.

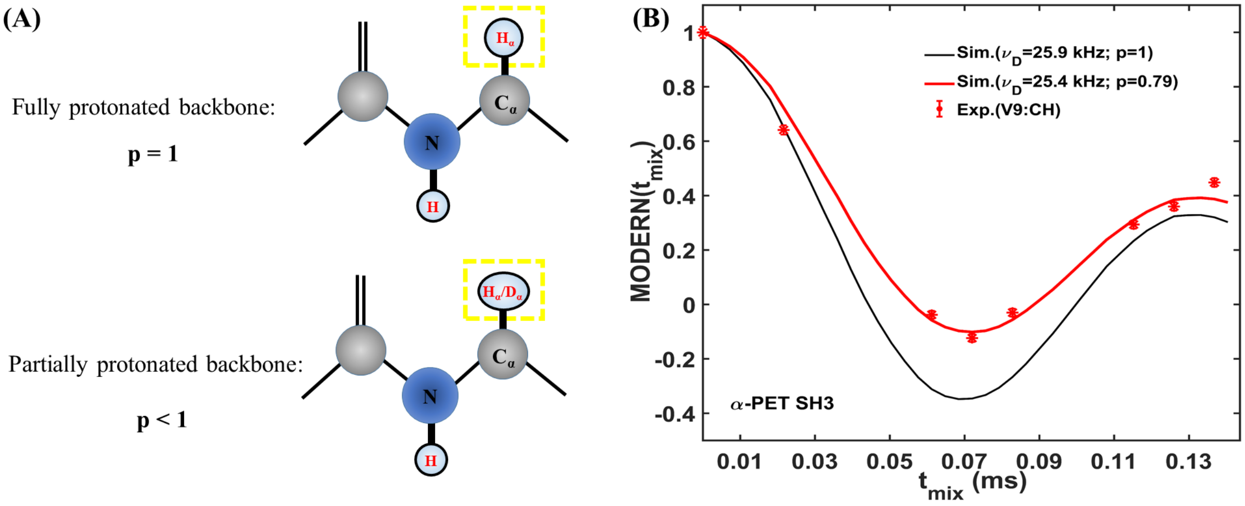

Figure S6. Partial protonation of backbone C_α carbons in α-PET SH3 sample. (A) The schematic presentation of two cases: all proteins with fully protonated backbones (upper, p=1) and some proteins (1-p, p<1) have deuterated

backbone (lower). (B) Experimental MODER5 V9 HC curve (red stars) and comparison of the simulated MODER5 curves (solid lines) with fully protonated backbone assumption (p=1, black line) and partially protonated backbone C_α carbon assumption (p=0.79, red line).

Figures S7 shows the experimental and simulated MODER5 curves for α-PET SH3 (long CP).

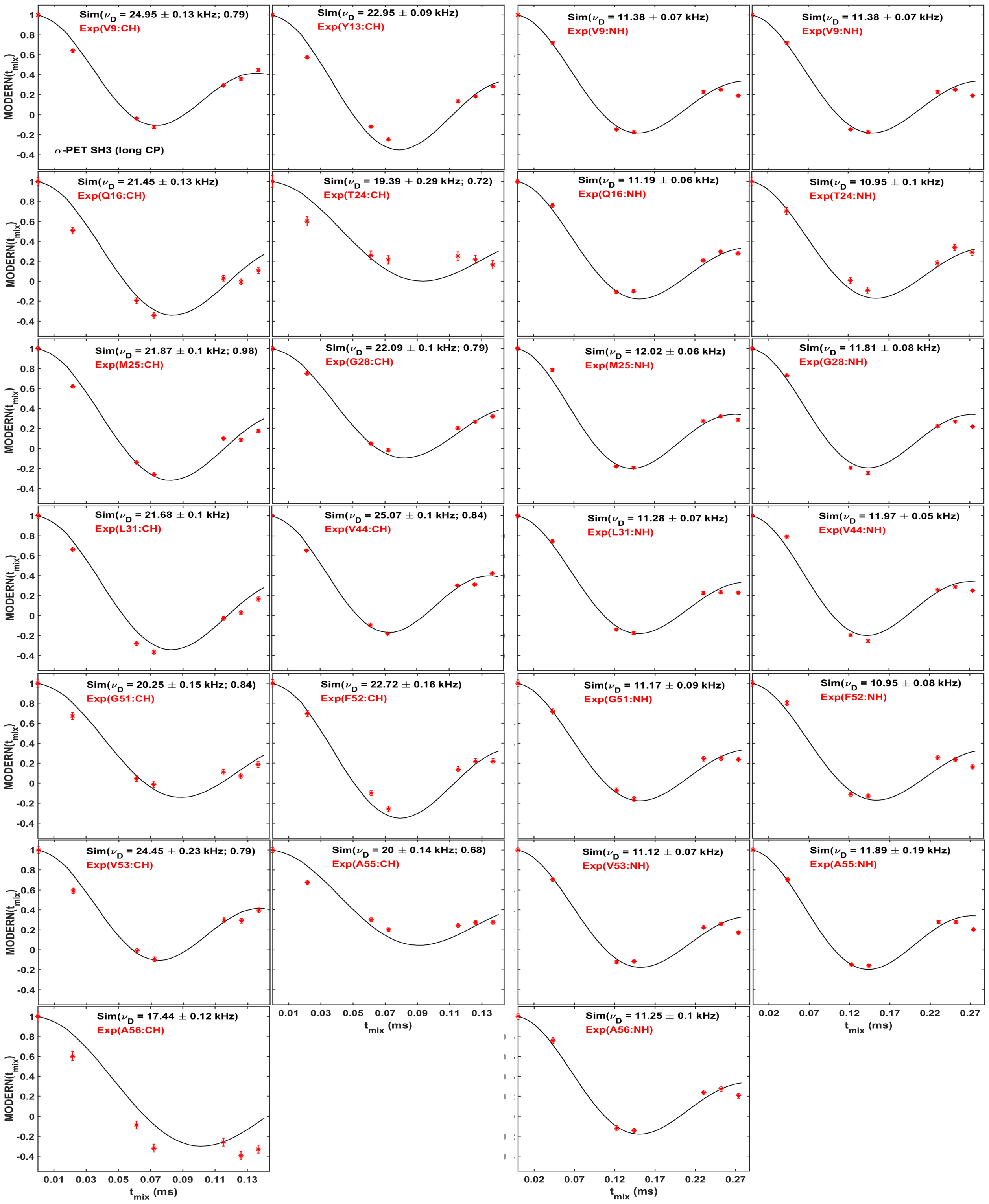

Figure S7. Experimental α-PET SH3 (long CP) and simulated MODER5 curves for HC (left two columns) and NH (right two columns) dipolar coupling values. The simulated MODER5 curves were obtained with $\Delta\alpha_{rf,i}$ and $T_{2,eff}$ (Table S1). Eqn. S4 represents the simulated signal. The fitting errors were obtained by generating 100 Monte Carlo curves, assuming the Gaussian noise distribution (~1.5σ).

Figure S8 shows the population of the protonated backbone C_α carbons for different residues. For some residues, C_α are fully protonated (Q16 and L31), whereas for others an apparent 35% of the signal comes from deuterated alpha carbon (T24).

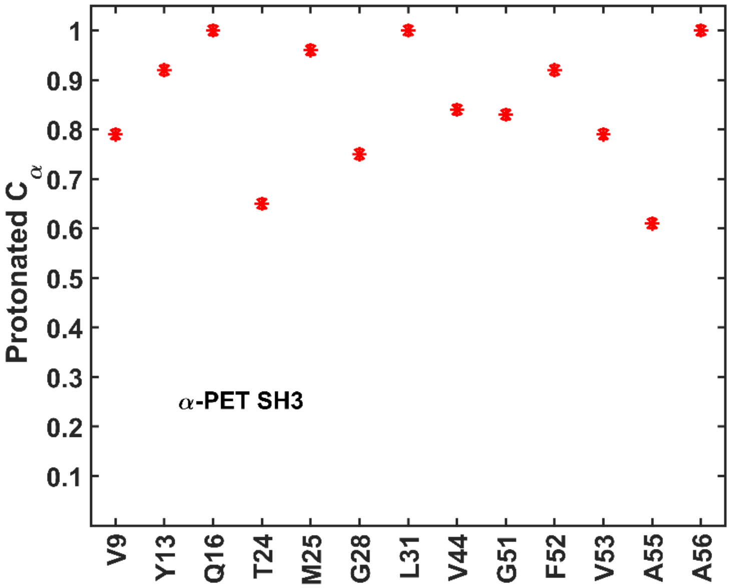

Figure S8. The fraction of signal arising from protonated backbone C_α carbons (p, axis Y) for different residues (axis X) for α-PET SH_3 sample. The population, p, was obtained from the comparison of experimental CH and simulated MODER5 curves.

Figure S9 summarizes the obtained HC (A) and NH (B) dipolar coupling values for all three samples. Additionally we compare NH dipolar coupling values, which were obtained by off-MAS method (cyan squares), of Xue *et al* (Ref. [8]).

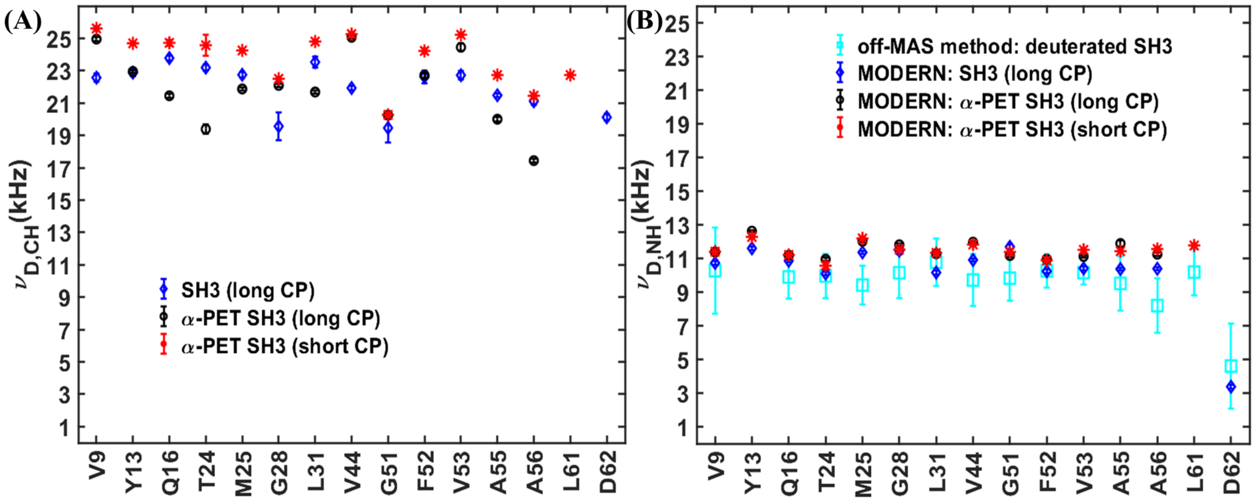

Figure S9. The obtained HC (A) and NH (B) dipolar coupling values for different samples: SH3 (long CP) – blue diamonds; α-PET SH3 (long CP) – black circles and α-PET SH3 (short CP) – red stars. In (B) cyan squares represent the NH dipolar coupling values, which were obtained by off-MAS method from deuterated SH3[8].

Torsion Angle Determination

The sequential application of MODERN pulses to recouple both HC and NH dipolar interactions allows measurement of the projection angle between these interactions, ϕ_{proj}. The projection angle can be visualized by translation of the HC bond to the NH bond such that C and N atoms coincide.

Figure S10 demonstrates the simulated dependence of MODER5-MODER5 curves on the projection angle for HC/NH (Figure S11A) and H_2C/NH (Figure S10B) groups. The dependence on the projection angle is shown between 0° and 90°, since the curves with ϕ_{proj} and 180°-ϕ_{proj} have almost identical behaviors at short mixing times. Comparing the simulated curves in Figure S10A with the curves in Figure S10B, it is clear that HC/NH curves are more sensitive to ϕ_{proj} than H_2C/NH curves, in particular below 0.05 ms. It shows the advantage of measuring the torsion angles with the deuterated α-PET SH3 sample, in which Cα of GLY residues (G28 and G51) contain only one proton and the second is replaced with deuterium.

Figure S11C shows simulated HC/NH curves, taking into account the experimental imperfections for α-PET SH3 with long CP ($\Delta\alpha_{rf,max} = 0.1$ and 0.55 ms of $T_{2,\mathrm{eff}}$). This Figure shows that the angle can still be precisely determined in the presence of RF inhomogeneity.

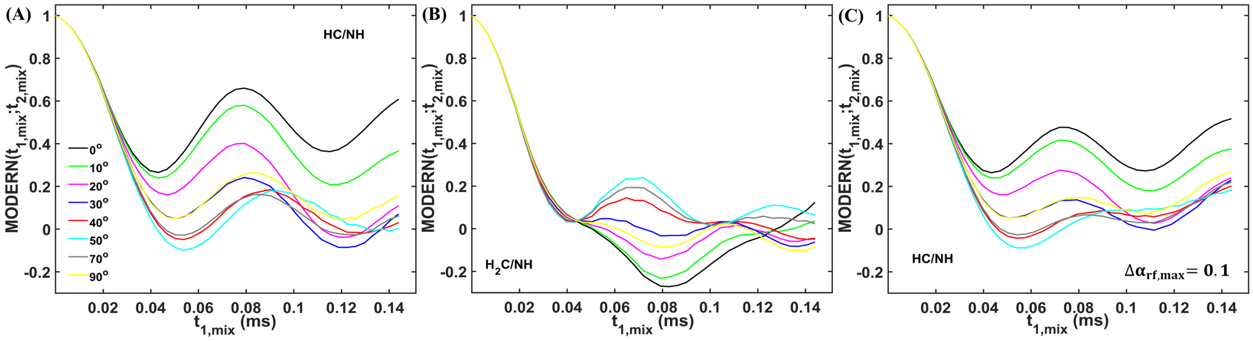

Figure S10. Simulated MODER5-MODER5 recoupling curves with different projection angle geometries (ϕ_{proj}). For HC and NH groups, D_{HC}=22 kHz and D_{HN}=11 kHz. The MAS rate is 55.555 kHz. The mixing time on NH dipolar pair ($t_{2,mix}$) is 2 times longer than on CH dipolar pair ($t_{1,mix}$): $t_{2,mix}=2t_{1,mix}$. (A) The projection angle between HC/NH with ideal MODER5-MODER5 simulations ($\Delta\alpha_{rf,max} = 0$). (B) The projection angle between H_2C-NH. The H-C-H angle is 109°. (C) The projection angle between HC/NH with $\Delta\alpha_{rf,max} = 0.1$.

However, the desired angle is a torsion angle, ϕ_H, which is produced by two planes defined by atoms Cα-N-H^N and Hα-C-N. The next Eqn. links the torsion and projection angles:

$$cos(\phi_{proj}) = sin(\theta_{HNC\alpha})sin(\theta'_{NC\alpha H\alpha})cos\phi_H + cos(\theta_{HNC\alpha})cos(\theta'_{NC\alpha H\alpha}), \qquad \text{Eqn. (S5)}$$

where $\theta_{HNC\alpha}$ is the H^N-N- Cα angle; $\theta'_{NC\alpha H\alpha} = 180^o - \theta_{NC\alpha H\alpha}$, where $\theta_{NC\alpha H\alpha}$ is the $NC_\alpha H_\alpha$ angle.

Figure S11 shows the conversion of the torsion angles, ϕ_H, between [0°:180°] into the projection angle domain for specific values of $\theta_{HNC\alpha}$ and $\theta'_{NC\alpha H\alpha}$. Figure S11A considers two cases: $\theta_{HNC\alpha} = 120^o$ whereas $\theta'_{NC\alpha H\alpha}$ is changed between 0° and 90° (blue area), and $\theta_{HNC\alpha}$ is changed between 90° and 180° whereas $\theta'_{NC\alpha H\alpha} = 71^o$ (red area). For example, when $\theta_{HNC\alpha} = 120^o$ and $\theta'_{NC\alpha H\alpha} = 0^o$, the projection angle is always 120° for all $\phi_H \in [0^o: 180^o]$. When $\theta_{HNC\alpha} = 120^o$ and $\theta'_{NC\alpha H\alpha} = 90^o$, the projection angles obtain values between [30°:150°]. In our case with $\theta_{HNC\alpha} = 120^o$ and $\theta'_{NC\alpha H\alpha} = 71^o$, the torsion angle range between [0°:180°] is converted into the projection angle range of [49:169].

Figure S11B shows the projection angle range, when $\theta'_{NC\alpha H\alpha}$ and $\theta_{HNC\alpha}$ are changed at the same time, while the difference $\theta_{HNC\alpha}$- $\theta'_{NC\alpha H\alpha} = 50^o$.

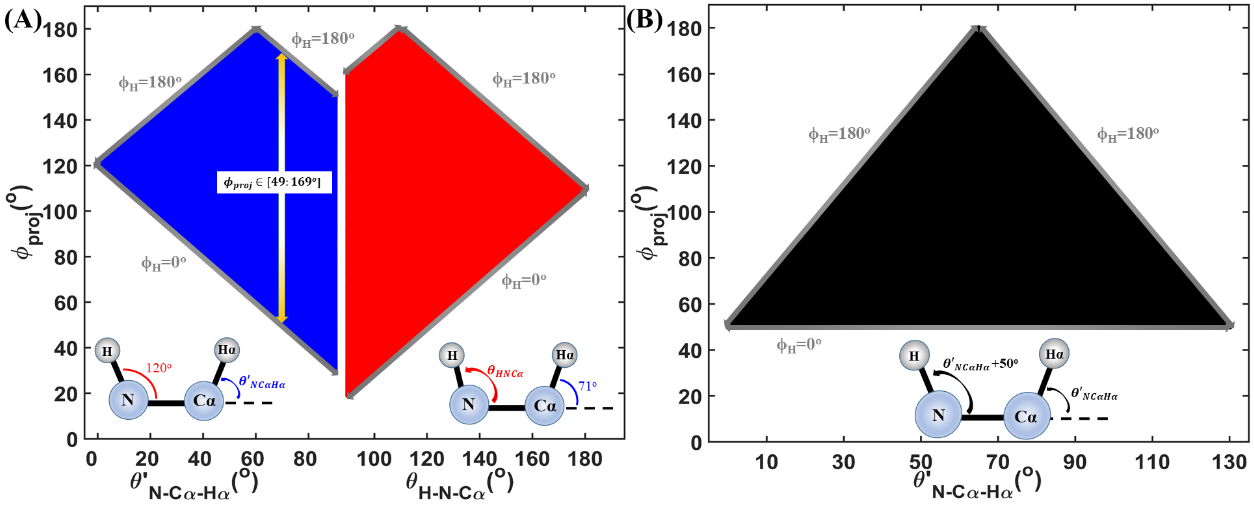

Figure S11. The dependence of the range of the projection angles, ϕ_{proj}, on $\theta_{HNC\alpha}$ and $\theta'_{NC\alpha H\alpha}$ values for $\phi_H \in [0^o: 180^o]$. (A) The projection angle range as a function of $\theta'_{NC\alpha H\alpha}$ (between 0° and 90°) with constant $\theta_{HNC\alpha} = 120^o$ (blue area) and as a function of $\theta_{HNC\alpha}$ (between 90° and 180°) with $\theta'_{NC\alpha H\alpha} = 71^o$. (B) The projection angle range as a function of $\theta'_{NC\alpha H\alpha}$ (between 0° and 130°), with $\theta_{HNC\alpha} - \theta'_{NC\alpha H\alpha} = 50^0$.

To obtain ϕ_H values we used the set of the HC, NH dipolar coupling values, $\Delta\alpha_{rf,max}$, and $T_{2,eff}$ as the input parameters. For torsion angle determination, ϕ_H value was the only fit variable. Figures S12-S14 show the comparison of the experimental and simulated MODER5-MODER5 curves for all three samples. Figure 12 also shows a plot of Eqn. S5.

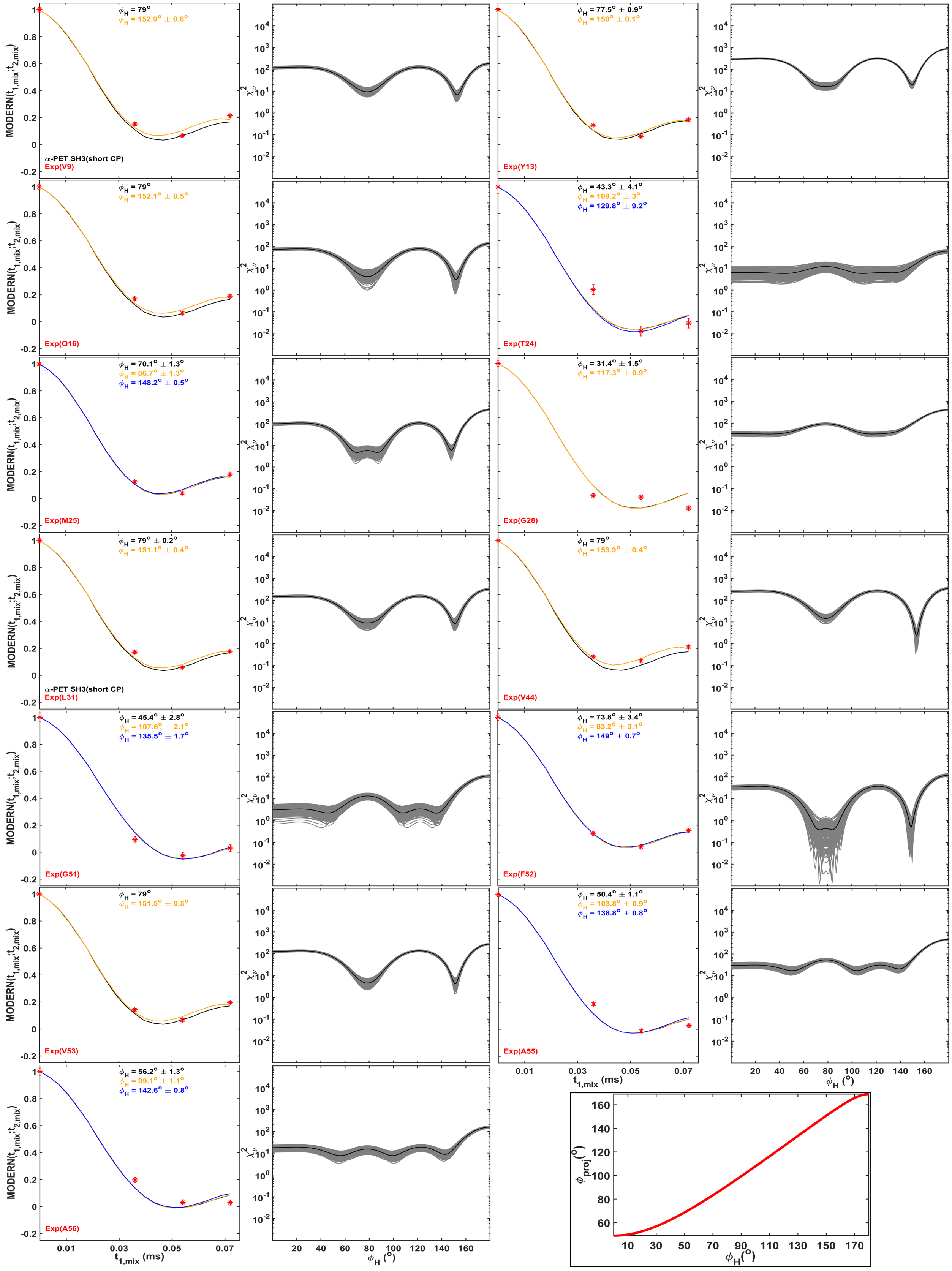

MODERN($t_{1,mix}$;$t_{2,mix}$)
χ^2_ν
α-PET SH3(short CP)
Exp(V9)
ϕ_H = 79°
ϕ_H = 152.9° ± 0.6°
Exp(Y13)
ϕ_H = 77.5° ± 0.9°
ϕ_H = 150° ± 0.1°
Exp(Q16)
ϕ_H = 79°
ϕ_H = 152.1° ± 0.5°
Exp(T24)
ϕ_H = 43.3° ± 4.1°
ϕ_H = 109.2° ± 3°
ϕ_H = 129.8° ± 9.2°
Exp(M25)
ϕ_H = 70.1° ± 1.3°
ϕ_H = 86.7° ± 1.3°
ϕ_H = 148.2° ± 0.5°
Exp(G28)
ϕ_H = 31.4° ± 1.5°
ϕ_H = 117.3° ± 0.9°
α-PET SH3(short CP)
Exp(L31)
ϕ_H = 79° ± 0.2°
ϕ_H = 151.1° ± 0.4°
Exp(V44)
ϕ_H = 79°
ϕ_H = 153.9° ± 0.4°
Exp(G51)
ϕ_H = 45.4° ± 2.8°
ϕ_H = 107.6° ± 2.1°
ϕ_H = 135.5° ± 1.7°
Exp(F52)
ϕ_H = 73.8° ± 3.4°
ϕ_H = 83.2° ± 3.1°
ϕ_H = 149° ± 0.7°
Exp(V53)
ϕ_H = 79°
ϕ_H = 151.5° ± 0.5°
Exp(A55)
ϕ_H = 50.4° ± 1.1°
ϕ_H = 103.8° ± 0.9°
ϕ_H = 138.8° ± 0.8°
Exp(A56)
ϕ_H = 56.2° ± 1.3°
ϕ_H = 99.1° ± 1.1°
ϕ_H = 142.6° ± 0.8°
$t_{1,mix}$ (ms)
ϕ_H (°)
ϕ_{proj}(°)

Figure S12. The first and third columns: Experimental α-PET SH3 (short CP) and simulated MODER5-MODER5 curves (solid lines). The second and fourth columns: χ_v^2(Eqn. S1) as a function of torsion angle, ϕ_H. The simulated MODER5-MODER5 curves were obtained with $\Delta\alpha_{rf,i}$ and $T_{2,eff}$ values of Table S1 and measured dipolar coupling values. The fitting errors were obtained by generating 300 Monte Carlo curves (grey curves, the black curve is the average), assuming a Gaussian noise distribution (~1.5σ) and considering the errors in the determination of dipolar coupling values. The Figure in the black frame shows the projection angle as a function of the torsion angle for $\theta'_{NC\alpha H\alpha} = 71^o$ and $\theta_{HNC\alpha} = 120^o$.

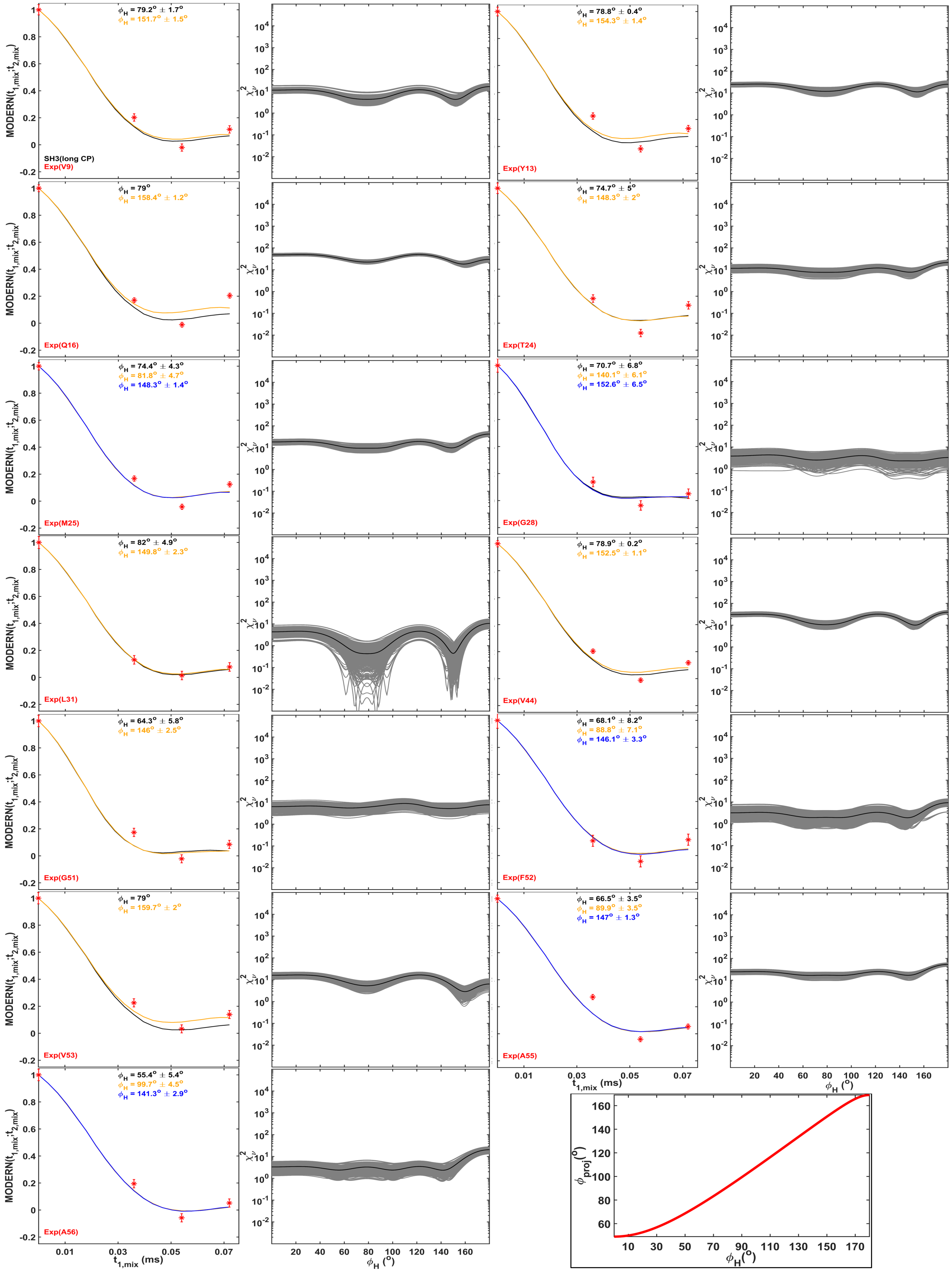

SH3(long CP)
Exp(V9)
ϕH = 79.2° ± 1.7°
ϕH = 151.7° ± 1.5°
Exp(Y13)
ϕH = 78.8° ± 0.4°
ϕH = 154.3° ± 1.4°
Exp(Q16)
ϕH = 79°
ϕH = 158.4° ± 1.2°
Exp(T24)
ϕH = 74.7° ± 5°
ϕH = 148.3° ± 2°
Exp(M25)
ϕH = 74.4° ± 4.3°
ϕH = 81.8° ± 4.7°
ϕH = 148.3° ± 1.4°
Exp(G28)
ϕH = 70.7° ± 6.8°
ϕH = 140.1° ± 6.1°
ϕH = 152.6° ± 6.5°
Exp(L31)
ϕH = 82° ± 4.9°
ϕH = 149.8° ± 2.3°
Exp(V44)
ϕH = 78.9° ± 0.2°
ϕH = 152.5° ± 1.1°
Exp(G51)
ϕH = 64.3° ± 5.8°
ϕH = 146° ± 2.5°
Exp(F52)
ϕH = 68.1° ± 8.2°
ϕH = 88.8° ± 7.1°
ϕH = 146.1° ± 3.3°
Exp(V53)
ϕH = 79°
ϕH = 159.7° ± 2°
Exp(A55)
ϕH = 66.5° ± 3.5°
ϕH = 89.9° ± 3.5°
ϕH = 147° ± 1.3°
Exp(A56)
ϕH = 55.4° ± 5.4°
ϕH = 99.7° ± 4.5°
ϕH = 141.3° ± 2.9°
MODERN(t1,mix;t2,mix)
t1,mix (ms)
χ²ν
ϕH (°)
ϕproj (°)

Figure S13. The first and third columns: Experimental SH3 (long CP) and simulated MODER5-MODER5 curves (solid lines). The second and fourth columns: χ_v^2(Eqn. S1) as a function of torsion angle, ϕ_H. The simulated MODER5-MODER5 curves were obtained with $\Delta\alpha_{rf,i}$ and $T_{2,eff}$ of Table S1 and measured dipolar coupling values. The fitting errors were obtained by generating 300 Monte Carlo curves (grey curves, the black curve is the average), assuming a Gaussian noise distribution (~1.5σ) and considering the errors in the measured dipolar coupling values. The Figure in the black frame shows the projection angle as a function of the torsion angle for $\theta'_{NC\alpha H\alpha} = 71^o$ and $\theta_{HNC\alpha} = 120^o$.

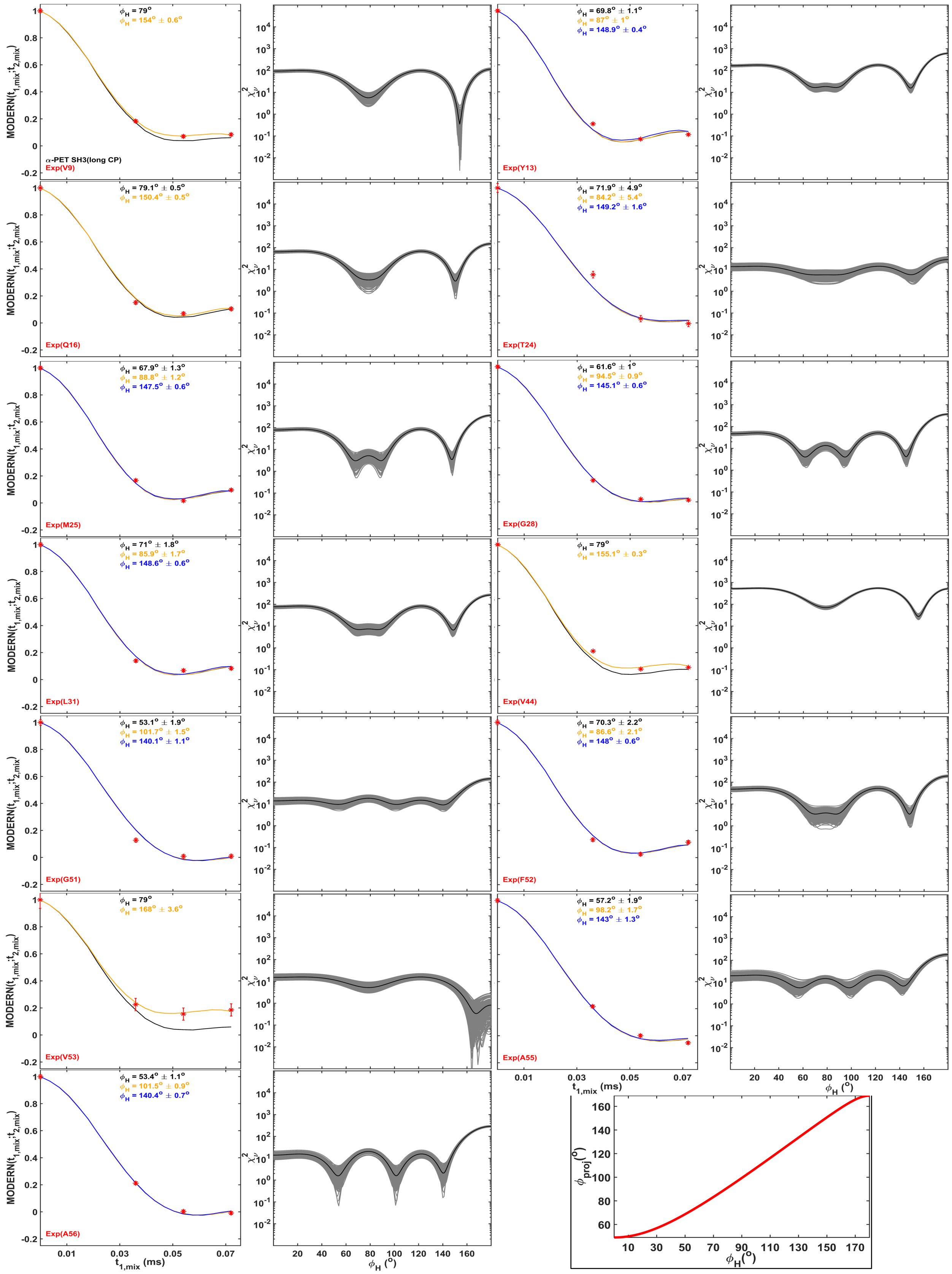
α-PET SH3(long CP)
Exp(V9)
ϕH = 79°
ϕH = 154° ± 0.6°
Exp(Y13)
ϕH = 69.8° ± 1.1°
ϕH = 87° ± 1°
ϕH = 148.9° ± 0.4°
Exp(Q16)
ϕH = 79.1° ± 0.5°
ϕH = 150.4° ± 0.5°
Exp(T24)
ϕH = 71.9° ± 4.9°
ϕH = 84.2° ± 5.4°
ϕH = 149.2° ± 1.6°
Exp(M25)
ϕH = 67.9° ± 1.3°
ϕH = 88.8° ± 1.2°
ϕH = 147.5° ± 0.6°
Exp(G28)
ϕH = 61.6° ± 1°
ϕH = 94.5° ± 0.9°
ϕH = 145.1° ± 0.6°
Exp(L31)
ϕH = 71° ± 1.8°
ϕH = 85.9° ± 1.7°
ϕH = 148.6° ± 0.6°
Exp(V44)
ϕH = 79°
ϕH = 155.1° ± 0.3°
Exp(G51)
ϕH = 53.1° ± 1.9°
ϕH = 101.7° ± 1.5°
ϕH = 140.1° ± 1.1°
Exp(F52)
ϕH = 70.3° ± 2.2°
ϕH = 86.6° ± 2.1°
ϕH = 148° ± 0.6°
Exp(V53)
ϕH = 79°
ϕH = 168° ± 3.6°
Exp(A55)
ϕH = 57.2° ± 1.9°
ϕH = 98.2° ± 1.7°
ϕH = 143° ± 1.3°
Exp(A56)
ϕH = 53.4° ± 1.1°
ϕH = 101.5° ± 0.9°
ϕH = 140.4° ± 0.7°
MODERN(t1,mix;t2,mix)
t1,mix (ms)
χ²ν
ϕH (°)
ϕproj (°)

Figure S14. The first and third columns: Experimental α-PET SH3 (long CP) and simulated MODER5-MODER5 curves (solid lines). The second and fourth columns: χ_v^2(Eqn. S1) as a function of torsion angle, ϕ_H. The simulated MODER5-MODER5 curves were obtained with $\Delta\alpha_{rf,i}$ and $T_{2,eff}$ values in Table S1 and measured dipolar coupling values. The fitting errors were obtained by generating 300 Monte Carlo curves (grey curves, the black curve is the average), assuming a Gaussian noise distribution (~1.5σ) and considering the errors in the measured dipolar coupling values. The Figure in the black frame shows the projection angle as a function of the torsion angle for $\theta'_{NC\alpha H\alpha} = 71^o$ and $\theta_{HNC\alpha} = 120^o$.

Figure S15A summarizes the obtained torsion angles as a function of the x-ray crystallography-derived angles from the pdb file 2NUZ. Figure S15B compares the absolute differences between the obtained torsion angles from α-PET SH3 using short CP and the x-ray crystallography-derived angles from pdb 2NUZ (red stars) and pdb 1SHG file (blue diamonds). These two structures have comparable unit cell parameters, but were reported independently from two different research groups. About a 5° rms deviation is observed between the two crystal structures. This can be compared with the 17.82° and 17.31° rms deviation from the NMR data.

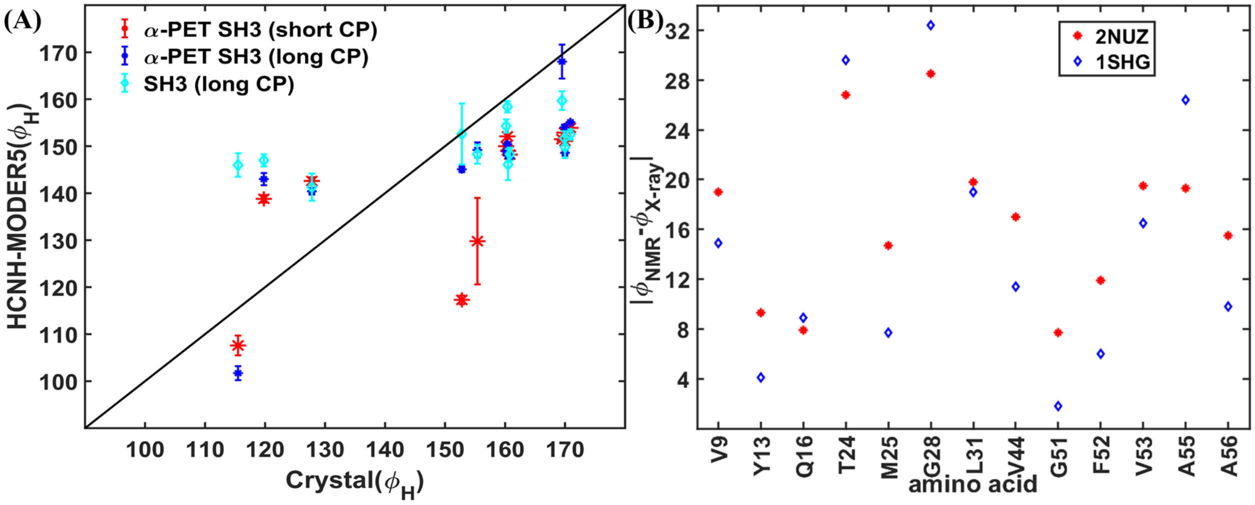

Figure S15. (A) Correlation of the torsion angles determined from X-ray crystallography (2NUZ file, axis x) and MAS NMR (axis y) methods. Red stars - α-PET SH3 (short CP) dataset; blue stars - α-PET SH3 (long CP) dataset and cyan diamonds - SH3 (long CP) dataset. (B) The absolute difference between the torsion angles from α-PET SH3 (short CP) sample and the torsion angles determined from 2NUZ file (red stars) and 1SHG file (blue diamonds).

Figure S16 demonstrates the experimental (red starts) and simulated (solid lines) MODER5 torsion angle curves of two residues W41 (A, C, E) and F47 (B, D, F) in three different M2 datasets and two samples, WT and S31N. M2 WT data was obtained with an 800 MHz magnet and 55.555 kHz MAS. The two other datasets (one for WT, one for S31N) were obtained with a 950 MHz magnet and 90.909 kHz MAS. The obtained torsion angles for different samples are in good agreement. The full experimental dataset for all resolved residues from the 950 MHz magnet is shown in Figures S18-S22.

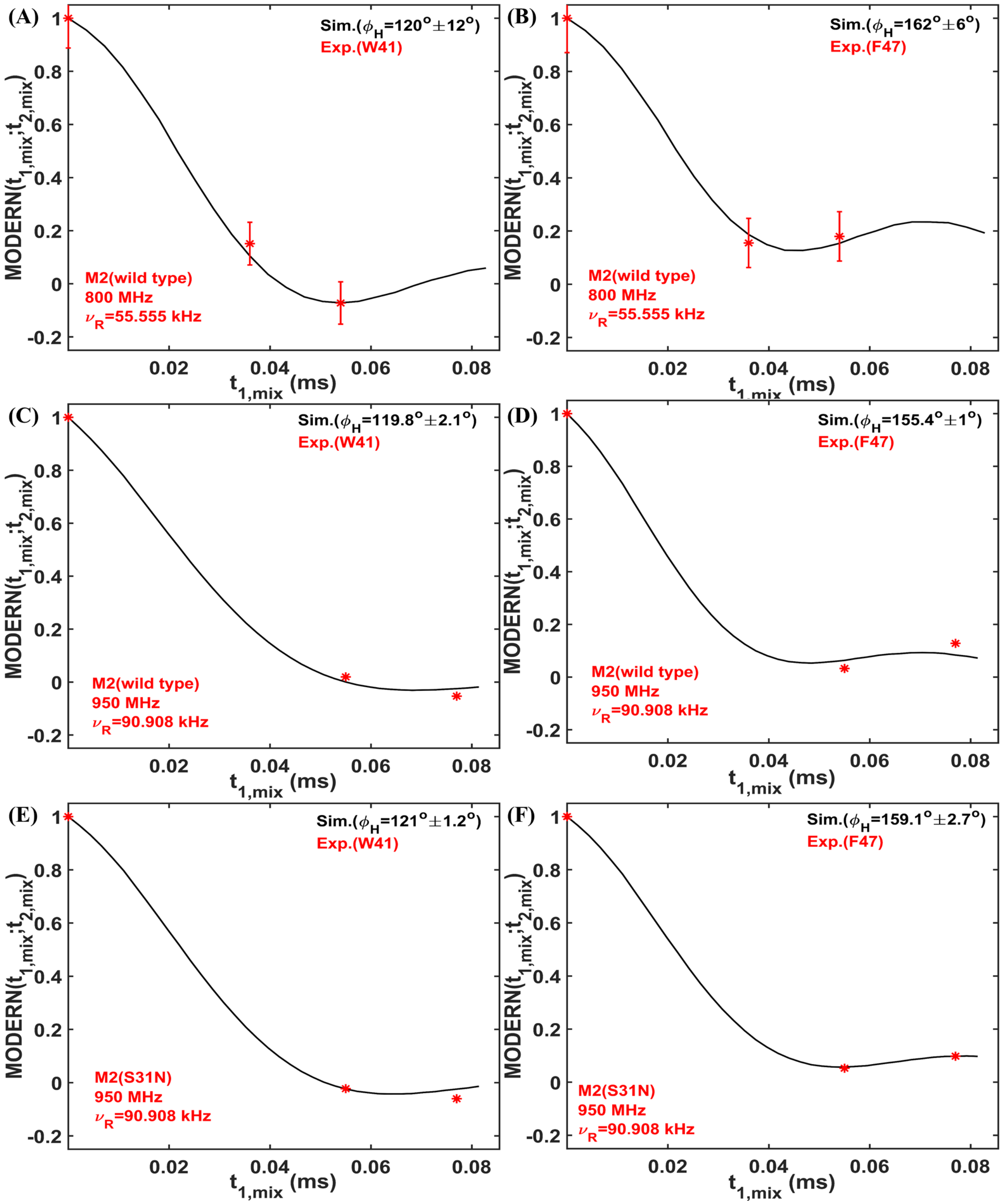

Figure S16. Best fit curves and torsion angle values for residues W41 (A, C, E) and F47 (B, D, F) obtained with HCαNH-MODER5 for M2 wild type (A, B, C, D) and M2 S31N (E, F). (A) and (B): Measurements were performed at an 800 MHz spectrometer using a 1.3 mm probe with 55.555 kHz MAS. (C)-(F): Measurements were performed at a 950 MHz spectrometer using a 0.7 mm probe with 90.908 kHz MAS. The data in Figures (A)-(B) and (C)-(F) were recorded as pseudo 4D and pseudo 3D experiments, respectively.

M2 data, 950 MHz spectrometer

This data was collected as pseudo 3D spectra at a 950 MHz spectrometer with 90.908 kHz MAS. Firstly, we optimized MODER5 rf-field power, in the similar way as for SH3 samples keeping short CP values. Additionally, we found that with high MAS rate, the incline of ramp of CP has a significant influence on the MODERN performance, as measured by the depth of the oscillation. Figure S17 demonstrates 1D detected MODER5 NH signal at 154 µs mixing for S31N M2 for four different sets of ramps. The best performance is observed with 90%→100% for HC CP and 70%→100% for NH CP (red). The starting signal (without mixing) varied by ~10 percent among these CP conditions. The same dependence was found for M2 wild type. Table S2 summarizes the optimized parameters: $\Delta\alpha_{rf,max}$ and $T_{2,eff}$.

Table S1 The obtained $\Delta\alpha_{rf,max}$ and $T_{2,eff}$ from comparison of three experimental NH MODERN curves (for each sample) with simulated curves.

sample	M2 (S31N)	M2 (wild type)
$\Delta\alpha_{rf,max}$	0.028	0.028
$T_{2,eff}$ (ms)	0.246±0.018	0.194±0.066

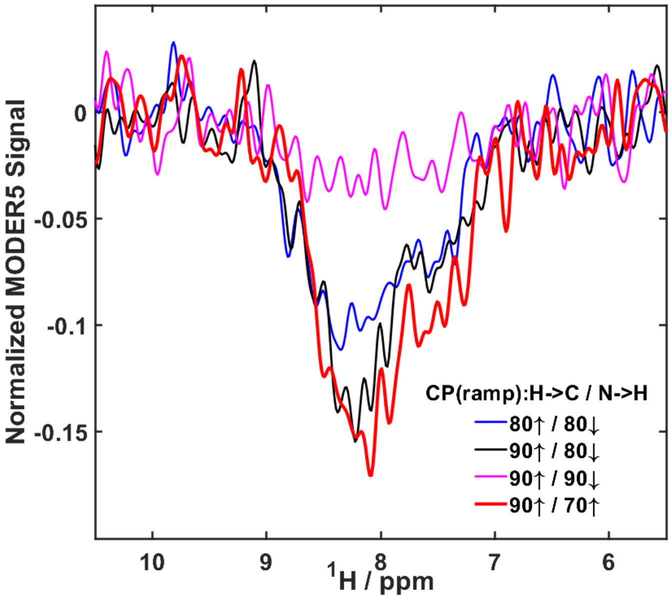

Figure S17. HN MODER5 optimization for M2 S31N at a spinning frequency of 90.908 kHz MAS. MODER5 HN signal (with 154 µs of MODER5 and optimized power level) for four different sets of ramped CP: blue – 80%→100% for CH and 100%→80% for NH; black – 90%→100% for CH and 100%→80% for NH; pink – 90%→100% for CH and 100%→90% for NH and red – 90%→100% for CH and 70%→100% for NH

Figures S18-S19 show the experimental and simulated MODER5 curves for M2 (wild type) and M2 (S31N), respectively.

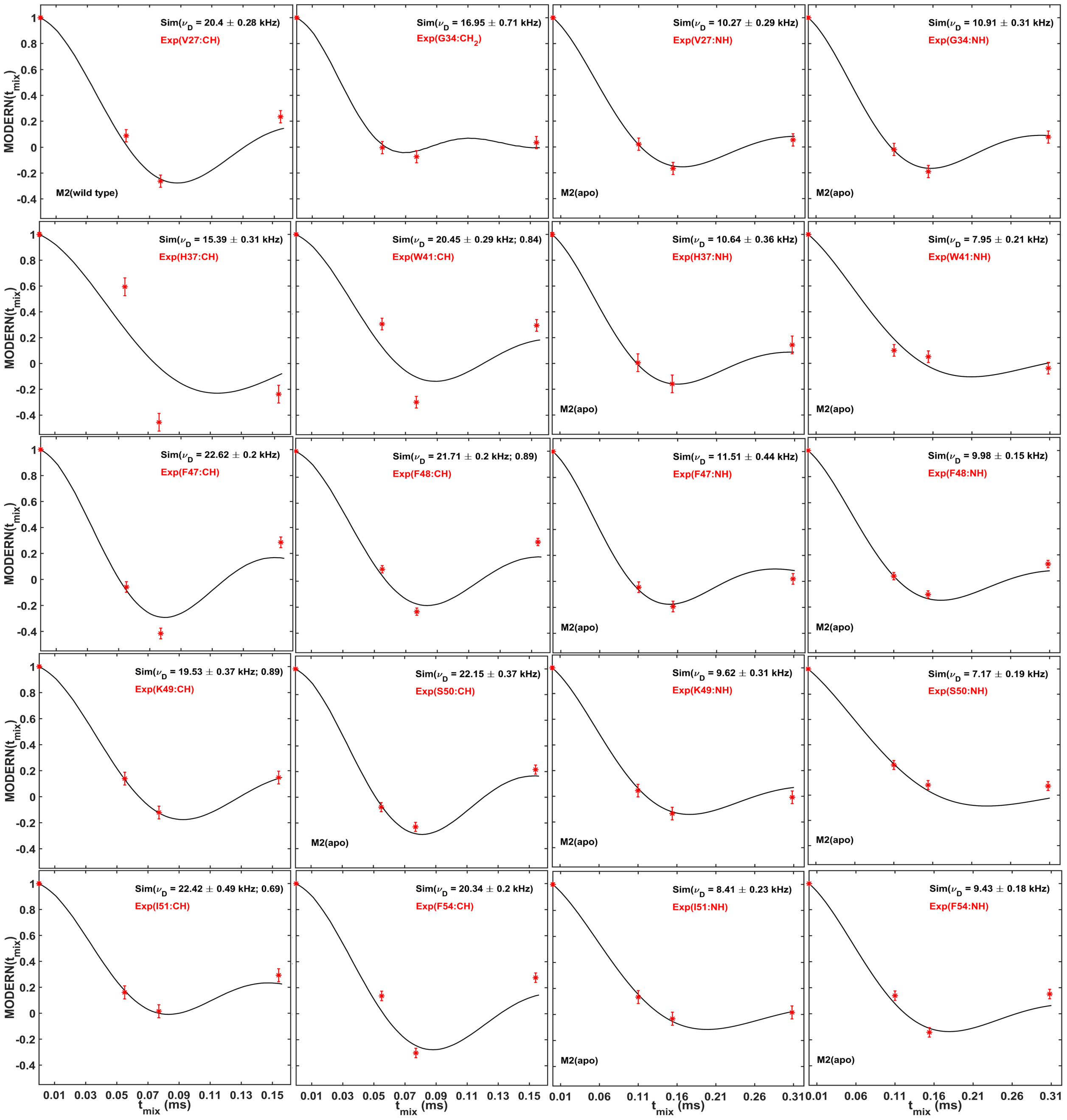

Figure S18. Experimental M2 (wild type) and simulated MODER5 curves for HC (left two columns) and NH (right two columns) dipolar coupling values. The simulated MODER5 curves were obtained with $\Delta\alpha_{rf,i}$ and $T_{2,eff}$ (Table S2). Eqn. S4 represents the simulated signal. The fitting errors were obtained by generating 100 Monte Carlo curves, assuming a Gaussian noise distribution. Error bars are displayed at 1.5σ. The dataset was recorded as pseudo 3D.

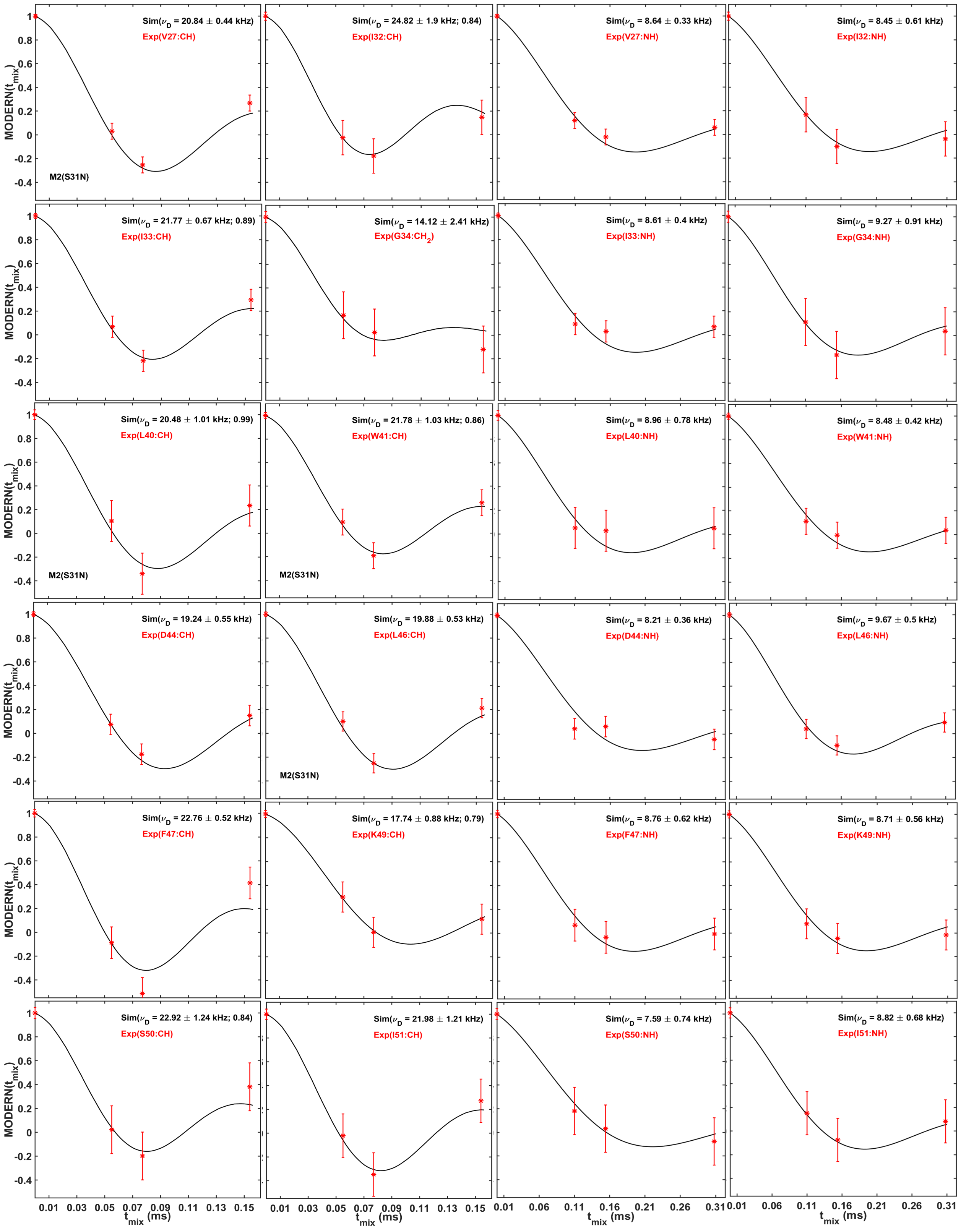

Figure S19. Experimental M2 (S31N) and simulated MODER5 curves for HC (left two columns) and NH (right two columns) dipolar coupling values. The simulated MODER5 curves were obtained with $\Delta\alpha_{rf,i}$ and $T_{2,eff}$ according to

Table S2. Eqn. S4 represents the simulated signal. The fitting errors were obtained by generating 100 Monte Carlo curves, assuming a Gaussian noise distribution. Error bars are displayed at 1.5σ. The dataset was recorded as pseudo 3D.

Figure S20 summarizes the obtained HC (A) and NH (B) dipolar coupling values. Figure C shows the population of C_αH pairs with a strongest dipolar coupling. Since this sample did not contain deuteration, the physical interpretation behind this population could be e.g. a small amount of overlapping signal from carbonyl resonances. At faster spinning, CP conditions can be broad enough for some carbonyl signal to survive the CP transfers, despite relatively selective CP conditions. In the pseudo-3D data, the carbon frequency is not sampled, such that carbonyl signal would overlap.

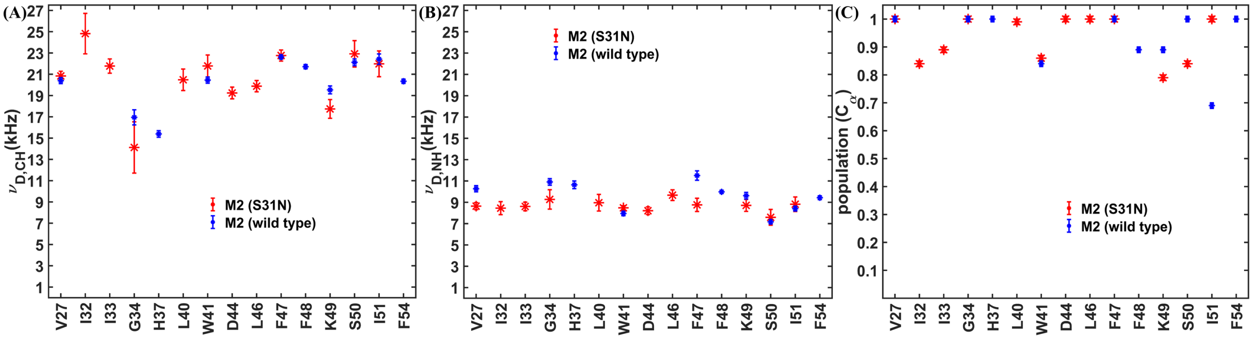

Figure S20. The obtained HC (A), NH (B) dipolar coupling and C_α population (C) values for different M2 samples: S31N – red stars and wild type – blue diamonds.

Figures S21-S22 show the comparison of the experimental and simulated MODER5-MODER5 curves for obtaining ϕ_H values.

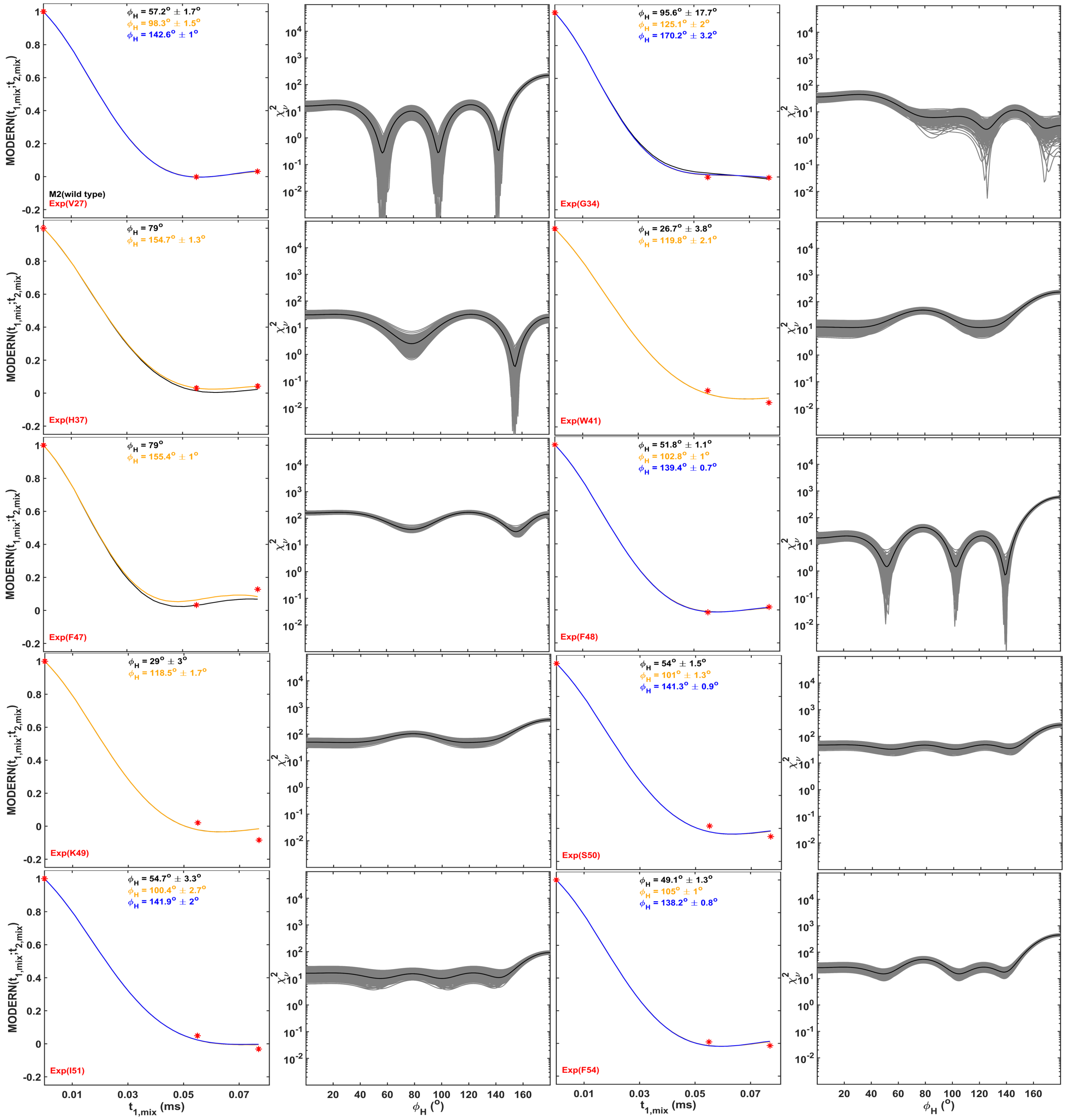

Figure S21. The first and third columns: Experimental M2 (wild type) and simulated MODER5-MODER5 curves (solid lines). The second and fourth columns: χ_ν^2(Eqn. S1) as a function of torsion angle, ϕ_H. The simulated MODER5-MODER5 curves were obtained with $\Delta\alpha_{rf,i}$ and $T_{2,eff}$ values of Table S2 and measured dipolar coupling values. The fitting errors were obtained by generating 300 Monte Carlo curves (grey curves, the black curve is the average), assuming a Gaussian noise distribution. and considering the error estimate in the determination of dipolar coupling values. Error bars are shown at 1.5σ.

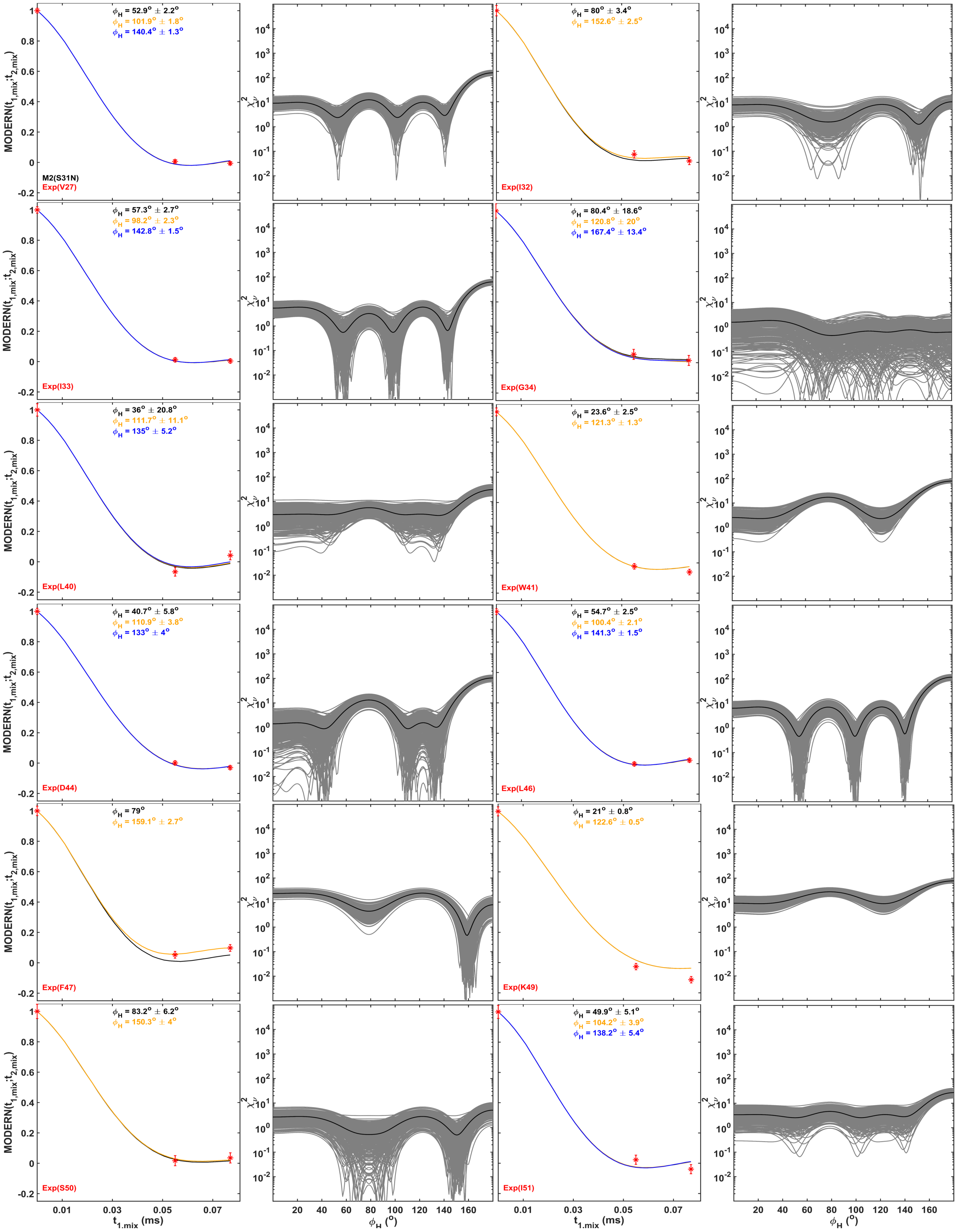

Figure S22. The first and third columns: Experimental M2 (S31N) and simulated MODER5-MODER5 curves (solid lines). The second and fourth columns: χ^2_ν(Eqn. S1) as a function of torsion angle, ϕ_H. The simulated MODER5-

MODER5 curves were obtained with $\Delta\alpha_{rf,i}$ and $T_{2,eff}$ values of Table S2 and measured dipolar coupling values. The fitting errors were obtained by generating 300 Monte Carlo curves (grey curves, the black curve is the average), assuming a Gaussian noise distribution and considering the errors in the determination of dipolar coupling values. Error bars are shown at 1.5σ.

Figure S23 compares the obtained ϕ_H values for each sample (red stars) with the TALOS predictions (black circles).

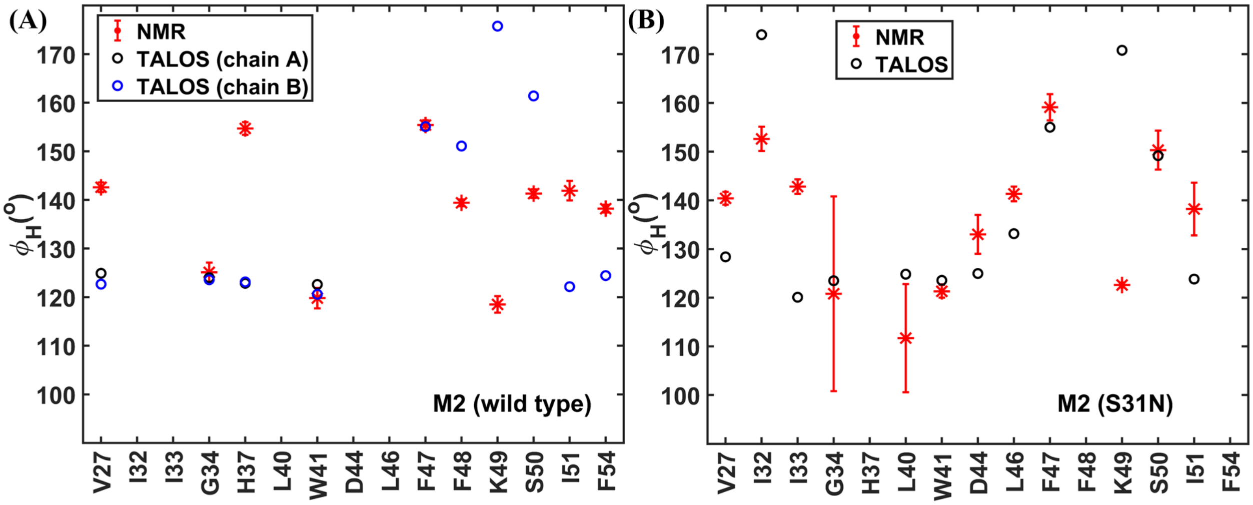

Figure S23. Comparison of the ϕ_H values which were obtained with NMR experiments (red stars) and predicted by TALOS (black and blue circles). (A) M2 (wild type) For TALOS calculations the isotropic chemical shift of chains A and B were used. (B) M2 (S31N). For NMR only one set of the possible values were used.

MATLAB Code

The MATLAB code is divided into three different MATLAB files: 'MODERN_SIMULATIONS.m', 'MODERN_D_Phi.m' and 'MODERN_Plot.m'. All input dipolar coupling values should be in kHz / the angles in degrees / the time as the number of MODERN blocks (1, 2, 3,… where one MODERN block consists of two 90° pulses). The first point is always t_{mix}=0.

<u>'MODERN_SIMULATIONS.m'</u> allows to simulate MODERN (dipolar coupling experiment, the Flag 'Dipolar_Torsion = 0', line 9) and MODERN-MODERN (torsion angle experiment, the Flag 'Dipolar_Torsion = 1', line 9). The dipolar coupling (D_C, line 11) or torsion angle (Phi_H, line 25) values can be inserted as an array (up to 10). Line 9 determines the increment number of MODERN blocks for the simulation of the dipolar interaction (jump_CH). The dipolar coupling values can be simulated for CH, CH2 or CH3 groups (the flag 'CH_flag_C', line 14). For the simulation of the torsion angles, the angle can be determined for HC-NH (CH_flag_C=1, CH_flag_N = 1, lines 14 and 24); H2C-NH (CH_flag_C=2, CH_flag_N = 1) or H2C-CH2 (CH_flag_C=2, CH_flag_N = 2). The MODERN curves can be simulated as ideal (da_rf_distr = 0, line 17) or with the influence of flip angle distribution and the effective relaxation: da_rf_distr = 1, line 17. The values da_rf_max (line 18) and T2_eff (line 19) are changed manually. The dependence of MODERN pulses can be simulated in the projection angle domain (The flag

‘projection_torsion_domain = 0’, line 26) or in the torsion angle domain (The flag ‘projection_torsion_domain = 1’, line 26). For the torsion angle domain, two additional angles are used: theta_NH (line 31) and theta_CH (line 32).

<u>‘MODERN_D_Phi.m’</u> code allows to obtain the dipolar coupling (The Flag ‘Dipolar_Torsion = 0’, line 11) and the torsion angle (The Flag ‘Dipolar_Torsion = 1’, line 11).
<u>‘MODERN_Plot.m’</u> code allows to plot the experimental and simulated signals with obtained dipolar couplings (The Flag ‘Dipolar_Torsion = 0’, line 8) and torsion angles (The Flag ‘Dipolar_Torsion = 1’, line 8).

‘MODERN_D_Phi.m’ code

<u>To obtain dipolar coupling values without $\Delta\alpha_{rf,max}$ and $T_{2,eff}$ optimization</u>:

‘Optimization_rf_T2=0’ (Flag at line 14)

‘Dipolar_Torsion = 0’ (Flag at line 25)

‘Exp_D.dat’ file should be prepared (line 36). This file can contain from 3 to 5 columns. The first column contains the numbers of applied MODERN blocks (minimal unit is 1, which defines a length corresponding to two 90°-pulses). The second column contains the experimental signal intensities. The third column contains the experimental errors. If some carbons have more than one directly bonded protons (CH2 or CH3 groups), the fourth column is added. For CH, CH2 and CH3 groups the numbers 1, 2 and 3 are added in this column, respectively. If some carbons are partially deuterated, the fifth column is added. For fully protonated cases, 1 is used, whereas for partially deuterated, 0.5. For example, ‘Exp_D_aPET_long_CP.dat’ file contains all five columns with CH, NH experimental data for V9. ‘Exp_D_aPET_short_CP.dat’ file contains only three columns. The ‘Exp_D_SH3_long_CP.dat’ file contains four, where CH2 and NH of G28 experimental signals are analyzed. The output file, ‘Dipolar values.dat’ contains the obtained dipole coupling values (the first row), and associated errors (the second row). For calculations of the dipolar coupling errors, the Monte Carlo curves are generated (The flag ‘montynumber = 100’, line 23). If the sample is partially deuterated, the third column represents the proton population obtained in the fit. For example, ‘Dipolar values.dat’ file contains the obtained CH dipolar coupling value of V9 residue and its population of the signal (0.79). As an example, Table S2 shows the input file for calculation of dipolar couplings, considering different populations and directly bonded protons. Two residues are shown, and the program recognizes the next residue by the 0 in the ‘Number of MODERN blocks’ column. .

Table S2. Example of the input file for dipolar coupling determination, consisting of 5 (maximal) columns. The first, second and third columns are the main columns – number of applied MODERN blocks, the experimental signal amplitudes and the errors. The fourth column defines the number of bonded protons (from 1 to 3). The fifth column defines the full protonation (1) or partial protonation (0.5). MODER5 CHn experimental data of two residues (G34 SH3 and V9 α-PET SH3 with long CP) are shown.

Number of MODERN blocks	Experimental signal	Experimental error	Number of bonded protons	Population
0	5015.706	306.2523	2	1
6	2787.737	306.2523	2	1
17	-347.9873	306.2523	2	1
20	-822.5881	306.2523	2	1
32	-240.2521	306.2523	2	1
35	-253.6302	306.2523	2	1
38	4.860873	306.2523	2	1
0	20667.95	296.603	1	0.5
6	13253.89	296.603	1	0.5
17	-789.4179	296.603	1	0.5
20	-2559.028	296.603	1	0.5
32	6071.143	296.603	1	0.5
35	7440.854	296.603	1	0.5
38	9266.049	296.603	1	0.5

To obtain dipolar coupling values with optimized $\Delta\alpha_{rf,max}$ and $T_{2,eff}$:

First Step

'Optimization_rf_T2=1' (Flag at line 25)

'Dipolar_Torsion = 0' (Flag at line 10)

The file 'T2_RF_OPT_EXP.dat' (line 44) should be prepared. It can contain any number of experimental signals. For example, 'T2_RF_OPT_EXP_aPET_short_CP.dat' file contains the NH experimental curves of four residues of α-PET SH3 (short CP): V9, Y13, A56 and L61. These experimental curves were chosen, since they had the longest MODER5 mixing times (for NH groups, the MODERN mixing time was two times longer than for CH group) and the normalized largest amplitudes were observed. The output file, 'opt_var.dat' contains the $\Delta\alpha_{rf,max}$ with the

error (the first row) and $T_{2,eff}$ (the second row). The example of output 'opt_var.dat' file for α-PET SH3 (long CP).

As an example, Table S3 shows an example input file for dipole coupling determination with optimization of flip angle distribution and $T_{2,eff}$.

Table S3. Example input file for the dipolar coupling values determination, consisting of 3 columns. The first, second and third columns are number of the applied MODERN blocks, the experimental signals and the errors. MODER5 NH experimental data of two residues (V9 and Y13 α-PET SH3 with short CP) are shown.

Number of MODERN blocks	Experimental signal	Experimental error
0	10636.37	279.719
12	7921.961	279.719
34	-2294.437	279.719
40	-3159.823	279.719
64	105.1874	279.719
70	1104.539	279.719
76	1803.188	279.719
0	19682.84	279.719
12	14210.3	279.719
34	-4998.771	279.719
40	-6647.696	279.719
64	2073.059	279.719
70	4034.151	279.719
76	5297.883	279.719

Table S4 shows the example of the output file with the optimal values.

Table S4. The example of the output file with optimal $\Delta\alpha_{rf,max}$ (the first row) and $T_{2,eff}$ (the second row) with the errors.

parameter	mean	std
$\Delta\alpha_{rf,max}$	0.028	0
$T_{2,eff}$ (ms)	0.648	0.233

Second Step

'Optimization_rf_T2=2' (Flag at line 25)

'Dipolar_Torsion = 0' (Flag at line 10)

'Exp_D.dat' file should be prepared in the same way as was described above.

In both cases, the output file, ‘Dipolar_values.dat’ file can contain two to three columns. The first two columns are the obtained dipolar coupling values and the errors. The third column represents the value of the protonation obtained in the fit (p≤1).

To obtain torsion angle values without $\Delta\alpha_{rf,max}$ and $T_{2,eff}$ optimization:

‘Optimization_rf_T2=0’ (Flag at line 25)

Dipolar_Torsion = 1’ (Flag at line 10)

The file ‘Exp_Phi.dat’ (line 48) should be prepared. It can contain from four to six columns. The first two columns define MODERN mixing times (number of blocks) for CH and NH groups, respectively. The third and fourth columns are experimental points and errors. The fifth column is added when some carbons have more than one directly bonded protons. The sixth column is added when the other spin can also have more than one directly bonded protons (for example, CH2-CH2). For example, ‘Exp_Phi_SH3_long_CP.dat’ file contains five columns for V9 and G28 residues, while ‘Exp_Phi_aPET_long_CP.dat’ file contains four columns. Additionally, the file “Dipolar_values_Phi.dat’ should be prepared. The first two columns should contain the obtained CH dipolar coupling values and their errors. The third and fourth columns should contain the obtained NH dipolar coupling values and their errors. The fifth column should be if some groups are partially deuterated. For example, ‘Dipolar_values_Phi_SH3_long_CP.dat’ file contains four columns, while ‘Dipolar_values_Phi_aPET_long_CP.dat’ file contains five columns. The comparison of the experimental and simulated data can be done with the projection angles (The flag ‘projection_torsion_domain = 0’, line 14) or in the torsion angle domain (The flag ‘projection_torsion_domain = 1’, line 14). For the torsion angle domain, two additional angles are used: theta_NH (line 19) and theta_CH (line 20).

The output files are two files. In the first, ‘phi_best.txt’ file, each row contains the obtained torsion angles with the errors. For calculations of the torsion angle errors, the Monte Carlo curves are generated (The flag ‘montynumber = 100’, line 23) and the errors of the dipolar coupling values are taken into account (three sets of the dipolar coupling values are simulated, the fit value and the fit value ± the estimated error at 1 standard deviation). The file, “aver_chi_curve.txt’ contains the averaged χ_ν^2 over 3*montynumber curves (montynumber – number of Monte Carlo curves, line 23) as the rows, whereas the file, “chi_curve_all.txt” contains all Monte Carlo curves as the rows.

‘MODERN_Plot.m’ code

To obtain dipolar coupling plots without $\Delta\alpha_{rf,max}$ and $T_{2,eff}$ optimization:

'Optimization_rf_T2=0' (Flag at line 21)

Dipolar_Torsion = 0' (Flag at line 9)

Additionally to the previous files, one more file should be created – 'Names.dat' (line 27). For example, 'Names_aPET_long_CP.dat' contains the names of V9:CH and V9:NH.

To obtain dipolar coupling plots with $\Delta\alpha_{rf,max}$ and $T_{2,eff}$ optimization:

'Optimization_rf_T2=1' (Flag at line 21)

Dipolar_Torsion = 0' (Flag at line 9)

The same file with the names of the residues should be prepared.

To obtain torsion angle plots without $\Delta\alpha_{rf,max}$ and $T_{2,eff}$ optimization:

'Optimization_rf_T2=0' (Flag at line 21)

'Dipolar_Torsion=1' (Flag at line 9)

Additionally to the previous files, one more file should be created – 'Names_Phi.dat' (line 27). For example, 'Names_Phi.dat' contains the names of V9 and G28.

To obtain dipolar coupling plots with $\Delta\alpha_{rf,max}$ and $T_{2,eff}$ optimization:

'Optimization_rf_T2=1' (Flag at line 21)

'Dipolar_Torsion = 1' (Flag at line 9)

The same file with the names of the residues should be prepared.

Table S2 summarizes the run time of the code per one experimental curve with specific flags, when full protonation is assumed. For partial deuteration, the time is increased about 10-fold (for 'Dipolar_Torsion' = 0 flag only). If the flip angle distribution is larger than 0.1, all run times are increased two-fold, since 14 MODERN curves are simulated and not 7.

Table S2 The time required to compare the simulated and the experimental MODERN signals and obtain dipolar coupling or torsion angle values. The calculations were performed on a desktop computer with an Intel® Core™ i5-7600 CPU @ 3.5GHz.

Optimization_rf_T2	0		1	2	
Dipolar_Torsion	0	1	---	0	1
Time (s)	~10	~140	~100	~70	~1200

Additionally to the mentioned input files (as examples), we provide a set of the input files, which can be used for running the codes (the experimental data of α-PET SH3 (short CP)):

'T2_RF_OPT_EXP.dat' (contains the experimental NH data of V9, Y13, A56 and L61 residues);

'Exp_D.dat' (contains the experimental CH data of V9, Y13, A56 residues and then the experimental NH data of V9, Y13, A56 residues);

'Exp_Phi.dat' (contains the experimental CH-NH data of V9, Y13, A56 residues);

‘NAMES.dat’ (contains the names of the residues for dipolar coupling value plots);
‘NAMES_Phi.dat’ (contains the names of the residues for torsion angle plots).

Sample preparation

α-PET SH3 and fully protonated SH3 samples were prepared according to the protocols in Refs. [9–11] and influenza A M2 protein, residues 18-60, was prepared according to the protocols in Refs. [12,13].

NMR Measurements

For NMR measurements at 600 MHz, either 0.3 ms (short CP) or 1.5 ms (long CP) H to C was applied, and 5.5 ms for N to C CP and 0.5 ms for N to H CP, in order to limit magnetization transfer to one bond. The MAS was 55 kHz in a 1.3 mm Bruker HXY wide-bore probe and the variable temperature (VT) gas was set to ~240 K. For measurements at 800 MHz, the short CP times listed above were used. The MAS was 55 kHz in a Bruker HCN narrow-bore probe and the variable VT gas was set to ~240 K. For measurements at 950 MHz, the short CP times listed above were used. The MAS was 90.908 kHz in a Bruker HCND narrow-bore probe and the VT gas was set to 255 K. The (H)CANH pulse sequence used as the basis for the torsion angle measurement was described in the publication of the Pintacuda group in 2014 (Ref. [14]) and the MODERN pulse sequence is according to Ref. [1].

1. Nimerovsky, E. & Soutar, C. P. A modification of γ-encoded RN symmetry pulses for increasing the scaling factor and more accurate measurements of the strong heteronuclear dipolar couplings. *J. Magn. Reson.* **319**, 106827 (2020).
2. Ahlner, A., Carlsson, M., Jonsson, B.-H. & Lundström, P. PINT: a software for integration of peak volumes and extraction of relaxation rates. *J. Biomol. NMR* **56**, 191–202 (2013).
3. Brinkmann, A. & Levitt, M. H. Symmetry principles in the nuclear magnetic resonance of spinning solids: Heteronuclear recoupling by generalized Hartmann–Hahn sequences. *J. Chem. Phys.* **115**, 357–384 (2001).

4. Zhao, X., Edén, M. & Levitt, M. H. Recoupling of heteronuclear dipolar interactions in solid-state NMR using symmetry-based pulse sequences. *Chem. Phys. Lett.* **342**, 353–361 (2001).
5. Paulson, E. K., Martin, R. W. & Zilm, K. W. Cross polarization, radio frequency field homogeneity, and circuit balancing in high field solid state NMR probes. *J. Magn. Reson.* **171**, 314–323 (2004).
6. Engelke, F. Electromagnetic wave compression and radio frequency homogeneity in NMR solenoidal coils: Computational approach. *Concepts Magn. Reson.* **15**, 129–155 (2002).
7. Gupta, R., Hou, G., Polenova, T. & Vega, A. J. RF INHOMOGENEITY AND HOW IT CONTROLS CPMAS. *Solid State Nucl. Magn. Reson.* **72**, 17–26 (2015).
8. Xue, K., Mühlbauer, M., Mamone, S., Sarkar, R. & Reif, B. Accurate Determination of 1H-15N Dipolar Couplings Using Inaccurate Settings of the Magic Angle in Solid-State NMR Spectroscopy. *Angew. Chem. Int. Ed.* **58**, 4286–4290 (2019).
9. Pauli, J., van Rossum, B., Förster, H., de Groot, H. J. M. & Oschkinat, H. Sample Optimization and Identification of Signal Patterns of Amino Acid Side Chains in 2D RFDR Spectra of the α-Spectrin SH3 Domain. *J. Magn. Reson.* **143**, 411–416 (2000).
10. Chevelkov, V. *et al.* Differential Line Broadening in MAS Solid-State NMR due to Dynamic Interference. *J. Am. Chem. Soc.* **129**, 10195–10200 (2007).
11. Movellan, K. T. *et al.* Alpha protons as NMR probes in deuterated proteins. *J. Biomol. Nmr* **73**, 81–91 (2019).
12. Schnell, J. R. & Chou, J. J. Structure and mechanism of the M2 proton channel of influenza A virus. *Nature* **451**, 591–595 (2008).
13. Andreas, L. B., Eddy, M. T., Pielak, R. M., Chou, J. & Griffin, R. G. Magic Angle Spinning NMR Investigation of Influenza A M218−60: Support for an Allosteric Mechanism of Inhibition. *J. Am. Chem. Soc.* **132**, 10958–10960 (2010).

14. Barbet-Massin, E. *et al.* Rapid Proton-Detected NMR Assignment for Proteins with Fast Magic Angle Spinning. *J. Am. Chem. Soc.* **136**, 12489–12497 (2014).
